# Core outcomes for the evaluation of new healthcare programmes – a modified Delphi study

**DOI:** 10.1186/s12913-025-12897-1

**Published:** 2025-05-27

**Authors:** Benjamin P. Harvey, Emmelie Barenfeld, Joakim Öhlén, Jana Bergholtz, Carl Johan Orre, Tomas Lindroth, Hanna Gyllensten

**Affiliations:** 1 Institute of Health and Care Sciences, Sahlgrenska Academy, University of Gothenburg, Box 457, Gothenburg, 405 30 Sweden; 2 University of Gothenburg Centre for Person-Centred Care (GPCC), Sahlgrenska Academy, Box 457, Gothenburg, 405 30 Sweden; 3 Institute of Neuroscience and Physiology, Department of Health and Rehabilitation, Sahlgrenska Academy, University of Gothenburg, Box 455, Gothenburg, 40530 Sweden; 4 Department of Computer Science and Media Technology, DVMT, Malmö University, Malmö, 211 19 Sweden; 5 Department of Applied Information Technology, University of Gothenburg, Box 100, Gothenburg, 41296 Sweden

**Keywords:** Delphi technique, Outcome and process assessment, healthcare, Consensus development, Healthcare economics and organizations, Patient-centred care, Person-centred care, Person-centered care, People-centred care, Centeredness, Decision making

## Abstract

**Background:**

New healthcare programmes that focus on the encounter between patients and healthcare services, such as those advocated for in different forms on “*centredness”,* are being pushed to the forefront of the healthcare agenda to, amongst other goals, combat rising costs. However, lack of consensus regarding which outcomes should be evaluated to cover the needs of all stakeholders creates barriers to prioritising between competing alternatives. The aim of this research was to develop a core outcome set (COS) for the evaluation of new healthcare programmes adopting centredness within the encounter between patients and healthcare services.

**Methods:**

A COS was developed according to the Core Outcome Measures in Effectiveness Trials guidelines, using the DelphiManager online platform. A list of outcomes was collected from literature, stakeholder group representatives and patient partners. The outcomes underwent a two-round modified Delphi method with representative groups including managerial decision-makers, researchers, health workers, and patients/patient representatives. Outcomes scored 7–9 (*critical)* by 70% or more participants and 1–3 (*limited importance)* by no more than 15% were deemed to have reached consensus at the conclusion of round two. The COS was finalised during the consensus meeting, conducted with patient partners and written input from stakeholder representatives.

**Results:**

An initial list of 65 outcomes from literature were refined to 51 items at the end of the pilot phase. At the completion of round two, eight outcomes had been scored critical by all stakeholder groups. A further 28 outcomes scored critical by at least one stakeholder group were included during the consensus meeting. The COS included 36 outcomes divided into six categories, i.e., general health, capabilities and support systems, care processes, organisational, economics, and eHealth.

**Conclusion:**

This was a first attempt at developing a COS for new healthcare programmes that adopt centredness within the encounter between patients and healthcare services, resulting in 36 outcomes divided into six categories judged critical by many of the included stakeholders. While providing a first step towards understanding stakeholder needs for implementing or participating in these healthcare programmes, variation between groups highlights the difficulties in allocating resources and attributing value to healthcare programmes practicing centredness.

**Patient and public involvement:**

Two patient partners formed an integral part of the research team by actively participating in the study design, participant recruitment, result analysis and writing of the final manuscript.

**Supplementary Information:**

The online version contains supplementary material available at 10.1186/s12913-025-12897-1.

## Background

Healthcare systems globally are facing significant challenges in providing high-quality care, such as an aging population, a greater number of people living with chronic conditions and comorbidities, growing consumer expectations, and an increase in technological advancements [1]. Addressing these challenges is further increased by financial constraints and the inefficient use of resources, forcing a rethink into how healthcare is organised and financed [1]. These are several of the drivers that have led to the advocation of healthcare programmes that adopt *centredness* within the encounter between patients (family, friends, informal carers: will be referred to as significant others henceforth) and healthcare services, including both the patient, health worker encounter and the patients overall experience with all provided healthcare services. These healthcare programmes promote the equal partnership between patients and health workers, whilst embracing individual values, needs and preferences, with the implementation of these programmes presented within a number of European initiatives as a solution to containing costs whilst still providing high levels of care [2–4].

The emergence of centredness in healthcare has been associated with the paternalistic shortcomings of conventional medical care, where the encounter between health workers and patients was perceived as too disease-oriented and failed to adequately address patients’ individual values, needs and preferences [5]. Patient-centred care was pioneering in shifting the attention to the patient’s perspective and acknowledging the “whole, unique person” [6]. Since its inception healthcare programmes adopting centredness have become ever more present within medical practice, policy and research, with several branches emerging, e.g. person-centred care, people-centred care, family-centred care. Despite small nuances between these healthcare programmes, Hughes *et.al. * [7] argues that at a conceptual level their similarities are greater than their differences, and common themes such as respect for individuality and values, shared responsibility, autonomy, therapeutic alliance and social context and relationships, could be used to characterise most forms of centredness [8, 9]. However, each of these themes are uniquely individual to each patient, healthcare service encounter, creating barriers to effectively measuring and evaluating their effectiveness on a group level. This requires that healthcare programmes practicing centredness have base standards for good practice, and methods to measure, monitor and evaluate [1]. This further extends to the increased implementation of eHealth initiatives within these healthcare programmes, with effective measurement and evaluation needed to account for the different digital modalities that facilitate these encounters.

The degree to which healthcare programmes adopting centeredness account for the aforementioned themes with suitable outcome measures is essential in evaluating and comparing their quality and effectiveness [4]. Consistency and reproducibility within research has seen an increase in the development and use of core outcome sets (COS), an agreed minimum set of outcomes that should be measured and reported in all clinical trials [10]. This has become more important as financial constraints place a greater focus on resource allocation and prioritisation, requiring healthcare programmes, such as those practicing centredness, to develop consistent methods for measuring and reporting [2]. Core outcome sets within this field are scarce, and focus primarily on patient-centred and person-centred interventions for diabetes, HIV and cancer, therefore tailoring their COS to address disease specific outcomes and not directly accounting for how healthcare services, and all involved actors, practice *centredness* [11–16]. Determining which outcomes should to be included when measuring centredness is a clear challenge, with initiatives aimed at collecting and categorising outcomes within important fields of practice, such as person-centred care, identifying 328 relevant patient-reported outcomes measures [17]. Through the standardisation of outcomes for healthcare programmes practicing centredness, a COS will improve the relevance of research findings to stakeholders with respect to the common themes that interlock these practices, allowing decision-makers to make informed choices regarding the implementation of these healthcare programmes [17, 18].

## Methods

### Aim

The aim of this research was to develop a COS for the evaluation of new healthcare programmes adopting centredness within the encounter between patients and healthcare services. This will allow for informed decision-making from all involved stakeholder groups in the design, implementation, and evaluation of such healthcare programmes.

### Study design

A modified Delphi (mDelphi) technique is defined by its use of subject matter ‘experts’ who provide information on group opinion through questionnaire rounds with controlled feedback mechanisms [19]. The methodology is considered advantageous because it ensures participant anonymity, avoiding the effect of dominant individuals, and can be circulated to large numbers with wide geographic dispersion [20]. In comparison to the “classical” Delphi technique, that enlists the opinions of stakeholders in the initial development of the outcomes, the “modified” Delphi technique begins consensus building from a more advanced starting point [21]. An analysis of current literature was succeeded by pilot interviews, two sequential questionnaire rounds and a final consensus meeting. The study was designed and reported in alignment with Core Outcome Measures in Effectiveness Trials (COMET) standards for development and reporting guidelines, the COS-STAD and COS-STAR, respectively [22, 23]. A detailed description of the process can be found in the study protocol (Supplementary file 1). The study was conducted using DelphiManager [24], an online platform hosted by Liverpool University.

### Context: the Swedish health system

Sweden has a universal health system that is largely tax-based, often referred to as the Beveridge model, with systems in place to reduce out-of-pocket costs for healthcare. Healthcare is organized (funded and predominately provided) by local regions and municipalities with a large degree of autonomy. However, irrespective of the provider, healthcare is expected to be evidence-based and prioritised according to an ethical platform based on principles for human dignity, needs and solidarity and the cost-effective use of recourses. While supporting a high life expectancy and quality medical care, critique towards the Swedish health system include a lack of accessibility and patient involvement [25]. The health system is currently undergoing a process to implement person-centred and integrated care to increase sustainability and cost-containment [26].

### Participants

Modified Delphi studies aimed at developing consensus should strive to include “experts” that are appropriate to the subject matter and are credible representatives of the target audience [27]. Four stakeholder groups were identified as key representative groups in the decision-making process regarding new healthcare programmes adopting centredness, managerial decision-makers, researchers, health workers, and patients/patient representatives. Eligibility criteria for each group was as follows; Managerial decision-makers were defined as responsible for the implementation decisions at state and national level, including government representatives and non-patient facing hospital managers. Health workers consisted of licensed physicians, nurses, physiotherapists, and occupational therapists. The patient/patient representative group was defined as having an illness for a minimum of 3 months or being involved in a patient advocacy group. Delphi panels are diversifying to include a broader range of expertise, included those with academic backgrounds [28]. Therefore, researchers, as the future users of the COS, were included and defined as conducting a doctoral education or higher, with sufficient knowledge of healthcare sciences.

### Patient and public involvement

Two patient partners formed an integral part of the research team, actively participating in each step of the project. One of the original patient partners ended their participation in the project before round one due to a worsening health status and was replaced. The overall aim of including patient partners within each step of the study was to use their knowledge to improve the quality and relevance of the research. The detailed steps for Patient and Public Involvement (PPI) are listed in the study protocol and include, for example, assistance in the recruitment of patient participants and the refinement of outcomes. The patient partners also acted as stakeholder group representatives for the patient/patient representative group, participating in the consensus meeting, as well as contributing to the manuscript (authors CJO, JB), and will assist with the dissemination of the published results. Patient and Public Involvement was reported in alignment with the Guidance for Reporting Involvement of Patients and the Public (GRIPP2) short form checklist [29].

### Data sources

**Fig. 1:**
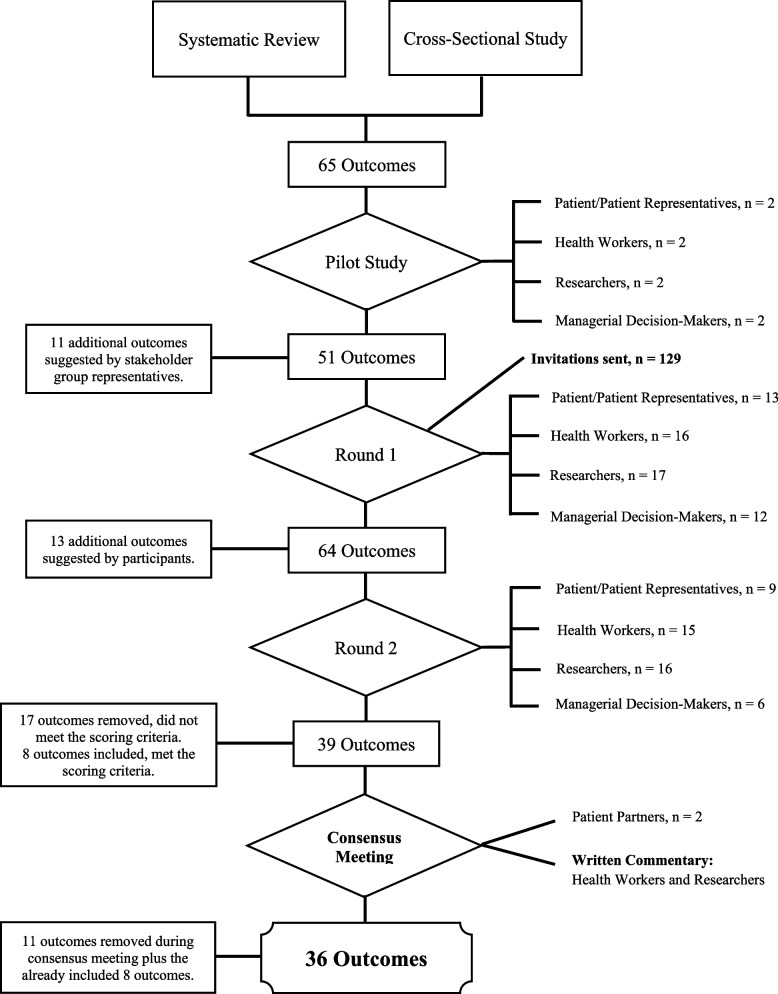
Modified Delphi process flow chart

Several information sources formed the initial list of outcomes that were piloted with stakeholder group representatives. Firstly, an ongoing systematic literature review (Prospero Registration #: CRD42022313047) evaluating both the cost-effectiveness and health equity of healthcare programmes that emphasis partnership between patients and healthcare services. Secondly, 163 separate outcome measures were extracted from a cross-sectional study [30] that analysed 27 interventional studies conducted by the Gothenburg Centre for Person-Centred Care. Patient partners also provided insight into suitable outcomes that were not identified within the aforementioned literature sources. A final list of 65 outcomes were assessed by the research team, duplicates removed, and the outcomes were translated into Swedish (Supplementary file 2). This first iteration of outcomes was piloted with stakeholder group representatives and patient partners creating a final list of 51 outcomes (Fig. 1). A description of the pilot phase can be found in the study protocol.

### Recruitment

The stakeholder group representatives and patient partners were identified based on recommendations from within the research group’s own network with regard to the aforementioned group eligibility criteria. They partook in the pilot phase, thereafter, providing a list of potential participants, who could later recommend further suitable candidates at the completion of the first questionnaire round, snowball sampling method [31]. In order to increase recruitment numbers, the research team, alongside patient partners, suggested possible candidates with the relevant expertise of their stakeholder group. Recommended participants were contacted via email and provided a detailed overview of the study, information on informed consent, and a link to DelphiManager. If a participant agreed to take part in the study, consent was given by logging in to DelphiManager and creating a profile. Each participant’s eligibility was assessed at the completion of round one to ensure they met the criteria of their stakeholder group. All participants that completed round one were eligible and included in the final results. An agreed sample size of approximately 15 participants per stakeholder group was deemed suitable by the research team and patient partners.

### Round one

Scoring in round one was available from the 26th of January 2023 until March 3rd, 2023. Each participant was provided approximately one month to complete the questionnaire consisting of demographic questions and the 51 outcomes. Participants were asked to score each outcome on a Likert-scale from one to nine (1–3 = limited importance, 4–6 = important, 7–9 = critical). An option was also provided to assign no score if the participant felt they lacked the appropriate knowledge or expertise to do so. Beside each outcome a text box was provided so that comments or feedback could be given regarding the individual outcomes. Participants were also provided an opportunity to suggest outcomes they believed were important and should be added to the questionnaire in the second round. Irrespective of scoring results, no outcomes were removed at the end of round one (Table S1).

### Round two

Scoring in round two was available from the 14th of April until May 27th, 2023. Prior to commencing round two the research team, alongside patient partners, evaluated any suggested outcomes and comments provided by the participants from the first round. Participants were once again asked to score the outcomes but were provided graphical scoring distributions of all stakeholder group’s scores from the first round [10]. Providing feedback to participants from all stakeholder groups has shown to improve consensus in terms of reduced variability in responses [32]. Any participant who changed their score between scoring thresholds (e.g. moving from 4–6 (important) to 7–9 (critical)), was provided with a text box to explain the reason for changing their score.

### Defining consensus

Consensus within mDelphi studies is measured based on the agreement or disagreement of an individual participant with a statement, later compared with group opinion and the extent to which participants agreed or disagreed with each other [10]. The method proposed for achieving consensus in this study is based on recommendations from the COMET initiative and those most used in research according to systematic reviews on Delphi methodology [22]. At the end of round two, the outcomes that had been scored 7 to 9 (critical) by 70% or more participants and 1 to 3 (limited importance) by no more than 15% were deemed to have reached consensus. Outcomes where consensus was not reached between all stakeholder groups were discussed in the consensus meeting. Remaining outcomes that were not scored critical by any stakeholder group were removed.

### Consensus meeting

The consensus meeting was held with two patient partners and two representatives from the research team that participated as moderators, alongside written commentary from the researcher and health worker stakeholder group representatives. Both managerial decision-maker representatives withdrew prior to the consensus meeting citing time constraints. In keeping with the COS-STAD guidelines, a criteria for including or excluding outcomes was defined prior to the consensus meeting [22]. Only those outcomes scored critical in round two by at least one stakeholder group were candidates for formal discussion, and required a majority consensus during the consensus meeting to be included in the COS.

### Ethical considerations

Selected participants scored outcomes based on their own views and opinions and therefore divulged no personal information about themselves during the study. One question requested sensitive information, “experience of having a long-term/chronic illness lasting longer than 3 months”, as it was regarded as important to understand if participants had experience of encountering healthcare services. Each participant’s identity was coded within DelphiManager, with the results being aggregated and presented at stakeholder group level. Therefore, no personal information was attached to the participants during the analysis or within the published results. Ethical approval (Dnr 2022–05445-01) was provided by the Swedish Ethical Review Authority.

## Results

### Round one

One hundred and twenty-nine people representing the four stakeholder groups were contacted via email and asked to participate in the study (Figure S1). Sixty-nine participants created a profile on DelphiManager of which 58 participants completed the questionnaire scoring 51 outcomes (Table 1). At the completion of round one all stakeholder groups had reached consensus on nine outcomes and a further 24 outcomes were scored critical by at least one stakeholder group (Table S2). Participants recommended 13 additional outcomes to be included in round two.

**Table 1:** Baseline demographics

Stakeholder Group	Patient/Patient Representative	Health Worker	Researcher	Managerial Decision-Maker	Total, n (%)
n (%)	18 (26%)	16 (24%)	21 (30%)	14 (20%)	69 (100%)
Sex (n)					
Male	8	4	6	5	23 (33%)
Female	10	12	15	9	46 (67%)
Age Group					
34 or younger	1	2	0	1	4 (6%)
35–44	1	8	4	3	16 (23%)
45–64	8	6	14	8	36 (52%)
65 or older	8	0	3	2	13 (19%)
Chronic Disease^a^					
Yes	14	1	5	7	27 (39%)
No	4	15	16	7	42 (61%)
Other Stakeholder Group^b^					
Patient/Patient Representative	5	0	1	0	6 (9%)
Healthcare worker	2	1	13	6	22 (32%)
Researcher	2	6	5	4	17 (25%)
Managerial Decision-Maker	5	3	0	0	8 (12%)
Informal carer	4	5	1	4	14 (20%)
None of the above	0	1	1	0	2 (3%)
Scoring Rounds					
Round 1 (n)					
Complete	13	16	17	12	58 (84%)
Incomplete	4	0	4	0	8 (12%)
Withdrew	1	0	0	2	3 (4%)
Round 2, (n)					
Complete	9	15	16	6	46 (79%)
Incomplete	3	1	1	6	11 (19%)
Withdrew	1	0	0	0	1 (2%)

*n* Number

^a^Do you have experience of having a chronic/long-term or serious disease/illness for a minimum of 3 months?

^b^Besides your initial reference group, do you identify with any of the other categories?

### Round two

Accounting for the 13 outcomes added at the conclusion of the round one, a total of 64 outcomes were scored or re-scored in round two (Table S3). Forty-six participants completed the questionnaire in round two. On 167 occasions participants changed their scores so that the outcome moved between scoring thresholds (Table S4). Seventeen outcomes were automatically removed after being scored critical by less than 70% of participants in all stakeholder groups. Eight outcomes were directly included in the COS, having been scored critical by more than 70% in all stakeholder groups, with no more than 15% scoring them as not important. Thirty-nine outcomes were scored critical by at least one stakeholder group.

### Consensus meeting and core outcomes

**Fig. 2:**
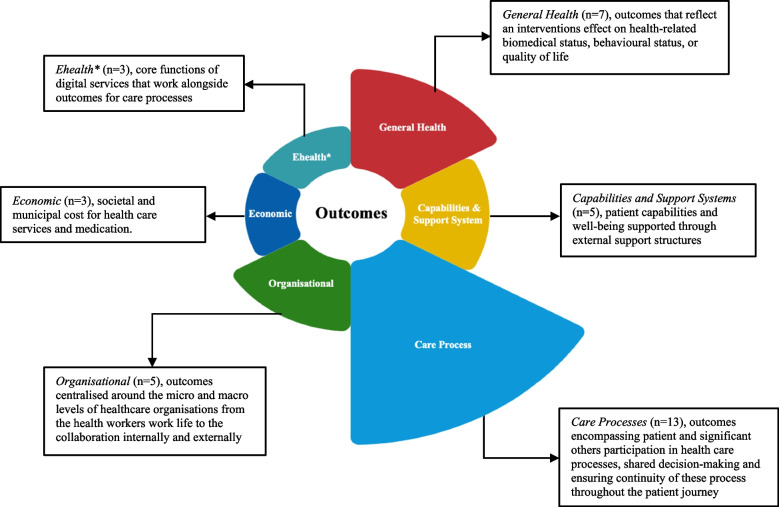
Core outcome set model. * Ehealth outcomes are applicable only to interventions that utilise an Ehealth medium

During the consensus meeting the representatives discussed 39 outcomes that reached consensus in at least one stakeholder group. The discussion was based on the scoring results from round 2 (Table S3), and written commentary from those stakeholder representatives that were not able to participate in the consensus meeting. A total of 28 outcomes deemed critical, resulting in a total 36 outcomes included in the final COS (Table 2). The outcomes were divided into six categories; 1) *General Health* (*n* = 7), outcomes that reflect an interventions effect on health-related biomedical status, behavioural status or quality of life; 2) *Capabilities and Support Systems* (*n* = 5), patient capabilities and well-being supported through external support structures; 3) *Care Processes* (*n* = 13), outcomes encompassing patient and significant others participation in healthcare processes, shared decision-making and ensuring continuity of these processes throughout the patients’ journey; 4) *Organisational* (*n* = 5), outcomes centralised around all organisational levels of healthcare, incorporating the health workers work life to the collaboration internally and externally; 5) *Economic* (*n* = 3), societal and municipal cost for healthcare services and medication; 6) *Ehealth* (*n* = 3), core functions of digital services that work alongside outcomes for care processes and are only applicable to interventions that have an eHealth intervention (Fig. 2).

**Table 2:** Final core outcome list

		Patient/Patient Representative (%)			Health Worker (%)			Researcher (%)			Managerial Decision-Maker (%)		
Category	Outcomes	1–3	4–6	7–9	1–3	4–6	7–9	1–3	4–6	7–9	1–3	4–6	7–9
General Health	Ability to perform personal activities in daily life	0	0	**100**	0	21.4	**78.6**	0	6.2	**93.8**	16.7	16.7	**66.7**
	Ability to work and study	11.1	0	**88.9**	6.7	40	**53.3**	0	18.8	**81.2**	16.7	66.7	**16.7**
	Ability to participate in social activities	0	11.1	**88.9**	0	26.7	**73.3**	0	12.5	**87.5**	16.7	33.3	**50**
	Pain/Discomfort	0	33.3	**66.7**	0	20	**80**	0	25	**75**	16.7	0	**83.3**
	Anxiety and/or Depression	0	28.6	**71.4**	0	26.7	**73.3**	0	12.5	**87.5**	0	16.7	**83.3**
	Patient's quality of life	0	0	**100**	0	0	**100**	0	0	**100**	0	0	**100**
	Significant others'quality of life	0	0	**100**	6.7	33.3	**60**	12.5	37.5	**50**	16.7	50	**33.3**
Capabilities and Support Systems	Ability to feel safe and secure	0	11.1	**88.9**	0	6.7	**93.3**	0	18.8	**81.2**	0	33.3	**66.7**
	Ability to experience one's life as meaningful	0	0	**100**	0	13.3	**86.7**	6.2	6.2	**87.5**	0	33.3	**66.7**
	Ability to effectively use coping strategies when faced with adversity	12.5	12.5	**75**	0	26.7	**73.3**	0	25	**75**	16.7	33.3	**50**
	Ability to experience love, friendship, and support	0	11.1	**88.9**	6.7	26.7	**66.7**	0	31.2	**68.8**	16.7	33.3	**50**
	Feeling of being seen as a person who is both capable and vulnerable	0	12.5	**87.5**	7.1	7.1	**85.7**	0	26.7	**73.3**	16.7	33.3	**50**
Care Processes	Preconditions to plan care based on the patient's lived experience and needs	0	0	**100**	0	26.7	**73.3**	0	12.5	**87.5**	0	33.3	**66.7**
	Preconditions to adapt planning based on the patient's needs	0	0	**100**	0	6.7	**93.3**	0	6.2	**93.8**	0	16.7	**83.3**
	Ability to adapt healthcare based on goals set together with the patient	0	11.1	**88.9**	0	13.3	**86.7**	0	6.2	**93.8**	0	0	**100**
	Conditions for the patient to have their questions and concerns addressed	0	11.1	**88.9**	0	6.7	**93.3**	0	12.5	**87.5**	0	16.7	**83.3**
	Conditions for significant others to have their questions and concerns addressed	0	11.1	**88.9**	0	26.7	**73.3**	0	43.8	**56.2**	16.7	50	**33.3**
	Preconditions for noticing changes in the patient’s needs	0	0	**100**	0	6.7	**93.3**	0	12.5	**87.5**	0	0	**100**
	Conditions of healthcare contributing to the patient having control over the continued care process	0	22.2	**77.8**	0	40	**60**	0	12.5	**87.5**	0	16.7	**83.3**
	Patient experience	11.1	11.1	**77.8**	0	0	**100**	0	12.5	**87.5**	0	33.3	**66.7**
	The patient feels they are treated with respect as an equal	11.1	11.1	**77.8**	6.7	6.7	**86.7**	0	13.3	**86.7**	0	33.3	**66.7**
	The patient's perspective and, if required, significant others'perspective is taken into account in all decisions regarding future care	0	11.1	**88.9**	13.3	13.3	**73.3**	0	31.2	**68.8**	0	16.7	**83.3**
	The patient and, if required, significant others are prepared for what is to come	0	11.1	**88.9**	0	6.7	**93.3**	0	12.5	**87.5**	0	0	**100**
	Continuity in health care	0	11.1	**88.9**	0	33.3	**66.7**	0	31.2	**68.8**	0	0	**100**
	Ability to see the unique individual	0	22.2	**77.8**	6.7	20	**73.3**	0	18.8	**81.2**	0	33.3	**66.7**
Organisational	Health worker's workload	11.1	33.3	**55.6**	0	13.3	**86.7**	0	37.5	**62.5**	0	16.7	**83.3**
	Health worker's work environment	0	55.6	**44.4**	0	13.3	**86.7**	0	18.8	**81.2**	0	0	**100**
	Health worker's ethical stress	0	55.6	**44.4**	0	13.3	**86.7**	0	18.8	**81.2**	0	16.7	**83.3**
	Healthcare's ability to collaborate externally	0	12.5	**87.5**	0	20	**80**	0	31.2	**68.8**	0	33.3	**66.7**
	Healthcare's ability to collaborate internally	0	0	**100**	0	6.7	**93.3**	0	12.5	**87.5**	0	16.7	**83.3**
Economics	Society's costs for healthcare	11.1	77.8	**11.1**	0	20	**80**	0	43.8	**56.2**	0	0	**100**
	Society's costs for medications	11.1	66.7	**22.2**	0	46.7	**53.3**	0	62.5	**37.5**	0	16.7	**83.3**
	Society's costs for municipal care (nursing homes and home help services)	11.1	44.4	**44.4**	0	57.1	**42.9**	0	56.2	**43.8**	0	16.7	**83.3**
eHealth	Accessible health information	11.1	33.3	**55.6**	0	53.3	**46.7**	0	31.2	**68.8**	0	0	**100**
	Experiencing services and functions as meaningful	0	11.1	**88.9**	0	13.3	**86.7**	0	31.2	**68.8**	0	16.7	**83.3**
	Experiencing the system as reliable	0	22.2	**77.8**	6.7	26.7	**66.7**	0	31.2	**68.8**	0	0	**100**

Bold indicates critical scores

### Results of patient and public involvement

The involvement of patient partners resulted in an increased number of participants being recruited to the study. Consultation also resulted in a broader and more diverse range of outcomes beyond the literature through accessing the patient partners’ lived experiences. Several questionnaire questions were also amended using lay language to ensure they were understandable to all involved stakeholder groups.

## Discussion

From an initial 65 outcomes identified within literature, eight outcomes were scored critical by all stakeholder groups at the conclusion of round two. A further 28 outcomes that were scored critical by at least one stakeholder group were included during the consensus meeting. The outcomes were grouped into six categories: general health, capabilities and support systems, care processes, organisational, economic, and eHealth. The final COS included 36 outcomes scored critical to the decision-making process for new healthcare programmes adopting centredness within the encounter between patients and healthcare services.

The category *general health* contains several outcomes that evaluate physical health and functioning, mental health, and quality of life. Previous COS [31, 33–35] have also acknowledged their importance when measuring centredness and incorporated these outcomes into their studies. General health captures many of the elements found in the EuroQoL (EQ-5D) questionnaire [33] used to calculate quality-adjusted life years (QALYs), converting the effects of an intervention into a single index measure to estimate the quantity and quality of life [34]. Despite being the most commonly used measure of health related quality of life in cost-effectiveness analyses, the QALY has been criticised for neutrality when assessing patients and does not adequately account for condition severity or health equity [35]. All outcomes found within the EQ-5D included in the COS were scored critical by the majority of stakeholder groups. It is also important to recognise that participants scored outcomes irrespective of diagnosis or condition, meaning individuals value healthcare programmes that address pain, depression, anxiety and performing activities in daily life, within all healthcare services. However, these outcomes account for only seven out of a total of 36, highlighting that despite being important to the decision-making process, attributing value and prioritising on these outcomes alone does not represent the preferences of stakeholders when assessing health programmes adopting centredness.

Attempting to move beyond the traditional methods of valuing healthcare programmes has seen the introduction of capability measures, assessing a healthcare programme’s influence on health-related functioning and well-being [36]. Well-being from a capability approach is captured through key elements such as stability, reflected in feeling settled and secure; attachment, through access to love and friendship; autonomy and control, as measures of independence; roles, representing opportunities to do things that make you feel valued; achievements, measured by life progress; and enjoyment [37]. Within the category *capabilities and support systems,* stakeholder groups scored five outcomes as critical, the ability to live a meaningful life, to feel safe and secure, to experience love and friendship, to effectively use coping strategies and to be seen as a person who is capable and vulnerable. The ability to live a meaningful life is unique within this category as the only outcome to be scored by 100% of patients, and more closely links with person-centred care ethics, separate from patient-centred research that emphasises functionality [8]. *Capabilities and support systems* are the first example in the COS where scoring differences between stakeholder groups appear, with the managerial decision-makers scoring all outcomes measures as non-critical. Not only does this demonstrate challenges in bridging the gap between macro, meso, and micro level decision-making, but it also highlights the difficulties in transitioning out of dominant paradigms when measuring the value of centredness [38].

Within taxpayer funded, universal healthcare systems, patients either receive free healthcare or incur a small out-of-pocket cost. However, patients are not always privy to the total costs related to their healthcare use [39]. Information asymmetry of this type can lead to an underappreciation of the true costs of healthcare and an overutilisation of services [40]. From the twelve outcomes defining various healthcare costs, only the managerial decision-maker group scored outcomes as critical, identify three to be included in the COS, society’s costs for healthcare, society’s costs for medication and society's costs for municipal care, including nursing homes and home help service. These results highlight a lack of transparency amongst all stakeholder groups with regard to costs, and could pose a number of problems for universal healthcare systems, such as moral hazard, where lower, or an absence of the knowledge of financial costs can lead to individuals exerting less effort in maintaining their health [41]. Mittler *et.al.* [42] identifies this as a performance-based argument for greater engagement of individuals in healthcare. This follows, that by removing obstacles, such as a lack of information, patients can make informed decisions that could lead to designing care plans and improving their overall health and well-being, potentially reducing costs. Increased transparency with respect to costs has been shown to reduce spending and could be a tool within universal healthcare systems to create a greater awareness regarding informed patient choice [40].

The category eHealth attempts to provide a key overview of stakeholder requirements when new healthcare programmes adopt digital modalities as a tool in achieving centredness. Three outcomes were regarded as critical by stakeholders, the availability of health information, experiencing services and functions as meaningful, and the reliability of eHealth systems. Challenges associated with assessing eHealth are that there are no standardised methods for measuring the benefits related to the impact on individual daily life [43]. The broad nature of the outcomes assessed with respect to eHealth can be regarded as a limitation of this study, however, it was beyond the scope of the research to adopt only an eHealth perspective. Additionally, the results reflect outcomes corresponding within the category “sense of security” that was identified by Lesén *et.al.* [43] who studied the preferential rankings of patient benefits of medical devices. When evaluating the effectiveness of centredness provided through an eHealth medium, all of the aforementioned categories and outcomes should be addressed, such as its impact on capabilities, or the degree to which it provides an inclusive environment for significant others and promotes efficient *care processes.*

At the conclusion of the study, three outcomes identified the role of significant others within *care processes* and highlighted that participation in healthcare decision-making needs to extend beyond the patient. The patient/patient representative group also scored “significant other’s quality of life” at 100% critical, whilst remaining stakeholder groups scored the outcome between 30%−60% as critical. Inclusion of this outcome was strongly argued for by the patient partners during the consensus meeting. According to Ekman *et.al.* [44] the economic value of informal care in Sweden is estimated at 150 billion Swedish Crowns annually, approximately 3% of the country’s gross domestic product. Understanding the broader societal impacts of illness is defined in economics as “family spillovers”, and despite a number of studies [45, 46] demonstrating its importance within economic evaluations, incorporating family spillover in economic evaluations is not common [47]. Several studies [12–14] that aimed to develop a patient-centred or person-centred COS within HIV, prostate and lung cancer did not evaluate or define outcomes specific to family members or informal caregivers. Acknowledging significant others is key within centredness research, however, this is not being reflected in the economic research or within the development of COS within this field.

## Strengths and limitations

Differences in the methodological approaches to COS development means that there are several potential sources of bias that can occur when developing and conducting the study [27]. Following the recommendations from the COMET initiative [27] the study protocol was prepared in advance, defining each step of the mDelphi process and outlining the methodological considerations to avoid potential biases. Firstly, the advanced starting point of a mDelphi risks that outcomes taken from literature may only be relevant to trialist and researchers [21]. Combining outcomes retrieved from literature with stakeholder interviews and including the involvement of patient partners, the outcomes reflect multiple perspectives, thereby increasing their relevance to broader population groups. Secondly, the mDelphi is advantageous as the anonymity of participants is preserved, reducing the influence of dominant personalities and allowing unfettered expression of opinions [48]. Individual communication with participants and personalised login details to DelphiManager protected participant anonymity and created space for open discourse when scoring outcomes. Finally, a formalised consensus meeting between stakeholders provided an avenue for discussing outcomes where consensus could not be reached. Although this makes the final COS subject to vulnerability with respect to typical group norms and values, it is regarded as essential in multi-stakeholder settings when attempting to reach consensus [27].

There are several limitations to the study that must be taken into consideration when interpreting the results of the COS, including attrition rates, outcome selection within the consensus meeting and deviations from the protocol. Sixty-nine people enrolled to take part in the study with 58 completing round one. This is in alignment with person-centred and patient-centred COS research that recruited participants ranging from 19 to 59 from multiple stakeholder groups [11, 12, 15, 16]. Williamson *et.al.* [27] states that by maintaining manageable group sizes, strategies can be adopted to increase response rates and combat high attrition rates, with a recommended acceptable response rate of approximately 80% within each stakeholder group. Both the patient/patient representative and the managerial decision-maker group had attrition rates significantly higher at the conclusion of round two, 50% and 57%, respectively. This was the main attributing factor to conducting two scoring rounds instead of three, as initial defined within the protocol. Understanding how this may bias the study results can be achieved by analysing the degree to which the results changed between rounds, did the participants who stayed have different views to those who left [49]. However, despite 27 participants changing their score in round two over 167 outcomes, only one outcome changed from being scored critical in round one, to not being scored critical by any stakeholders in round two. Five outcomes that were not scored critical in round one, were scored critical in round two.

Furthermore, several challenges were encountered with respect to recruitment and attrition of the managerial decision-maker stakeholder group and stakeholder representatives. As initially defined within the protocol, this stakeholder group was called “Decision-makers”, with the aim of recruiting politicians. Changes were made to the definition of this group citing that no politicians could be recruited, and therefore “Managerial decision-makers” was created focusing on the recruited government representatives and non-patient facing healthcare managers. Not only did this scoring group encounter the largest attrition rates, both stakeholder group representatives withdrew from the study prior to the consensus meeting, thereby not advocating for or against the scoring decisions. Attrition of stakeholder representatives prior to the consensus meeting could potentially bias the final results and lead to a COS that is not representative of all the stakeholder groups that were identified as key actors in decision-making with regard to these new healthcare programmes.

Finally, this mDelphi study was conducted within Sweden and therefore the perspective of the different stakeholder groups may not adequately represent an international perspective with respect to the healthcare costs and utilisation outside of those countries with universal healthcare systems. However, outcomes that encompass the other categories within the COS have been collected and translated from international sources, highlighting their importance within healthcare services globally. Additionally, generalisability is enhanced within COS that incorporate a diverse array of stakeholder perspectives such as patients/patient representatives, researchers, health workers, managers [50].

## Patient and public involvement

The contribution of patient partners as both members of the research team and stakeholder group representatives has led to a broader incorporation of outcomes that captured the perspective of the patient’s own involvement within healthcare services adopting centredness. Within the design phase, this can be summed up by outcomes that begin with the phrase “the patient feels” as if they have been treated or recognized in a specific manner. Patient partners addressed this by highlighting that is not merely enough for healthcare service to say that they adopt centredness, but that patients and significant others need to feel that this was the case. The incorporation of patient partners is not without its challenges, as experienced within the consensus meeting, with two outcomes, significant others’ quality of life and the ability to experience love, friendship, and support, being included despite all other stakeholders scoring these as non-critical. Alongside the attrition of stakeholder representatives before the consensus meeting, this creates a risk that, without the same passionate defence of the outcomes by other stakeholder representatives, the COS is favourable to the patient perspective and may not adequately account of all other perspectives equally.

## Suggestions for future research

The outcomes that have been collected and scored in this mDelphi study require further exploration and development in order to be practically applicable in evaluating healthcare programmes that adopt centredness. Despite many of the patient-reported outcomes being identified within validated measurement instruments, e.g., EQ-5D, ICEpop CAPability measure, Person-centred Care Assessment Tool, there are still some that, to the best of our knowledge, are not found within current, validated questionnaires. Additionally, although several outcomes were identified in the aforementioned outcome measures, instead of attempting to develop a single questionnaire, the outcomes require division and allocation with respect to their appropriate target respondent in order to define an overarching research process in its entirety. An example of this is that outcomes encompassing workplace stress need to be answered by health workers, economic outcomes, on many occasions, will be addressed by researchers through registries. Those outcomes that are defined as suitable for patient reported outcome measures also require prioritisation, possibly through discrete choice experiments, in order to obtain weighted scores for each outcome for the purpose of measuring the results of future questionnaire studies. This also requires conducting similar studies on larger population groups throughout multiple countries and healthcare settings.

## Conclusion

This was a first attempt in developing a COS for the evaluation of new healthcare programmes that adopt centredness within the encounter between patients and healthcare services, resulting in a list of 36 outcomes, divided into six main categories judged critical by the included stakeholder groups. Although this is a first step in understanding stakeholder needs for participating in, and evaluating centredness, the differing opinions on the perceived importance of significant others, capabilities measures, and healthcare costs, indicate that further research is needed to understand and meet individual needs, and inform policy about the true benefits of these healthcare programmes.

## Supplementary Information

Supplementary Material 1.

## Data Availability

The datasets generated and analysed during the current study are not publicly available because they contain information that can compromise research participants’ privacy/consent. Such data can only be made available, after legal review, to researchers who meet the criteria to access data, according to the General Data Protection Regulation, the Swedish Data Protection Act, the Swedish Ethical Review Act, and the Swedish Public Access to Information and Secrecy Act. Readers may contact Benjamin P. Harvey (benjamin.peter.harvey@gu.se) regarding these data.

## Abbreviations

COS: Core Outcome Set
mDelphi: Modified Delphi
COMET: Core Outcome Measures in Effectiveness Trials
PPI: Patient and Public Involvement
GRIPP2: Guidance for Reporting Involvement of Patients and the Public (GRIPP2) short form
EQ5D: EuroQoL Questionnaire
QALY: Quality Adjusted Life Years
