# Supplementary material for: Core outcomes for the evaluation of new healthcare programmes – a modified Delphi study
Source: BMC Health Serv Res. 2025 May 27;25:758. doi: 10.1186/s12913-025-12897-1 (PMC12107808; doi:10.1186/s12913-025-12897-1)
Supplement: Supplementary file 1 — Supplementary Material 1. [file 12913_2025_12897_MOESM1_ESM.docx]

A Core Outcome Set for the Evaluation of New Healthcare Programmes – A Modified Delphi Study

Authors: Harvey B. P ^1,2^, Barenfeld E ^2,3^, Öhlén J ^1,2,4^, Bergholtz J ^1,2,5^, Orre C. J ^6^, Lindroth T ^2,7^, Gyllensten H ^1,2^

^1^ Institute of Health and Care Sciences, University of Gothenburg, Box 457, 405 30 Gothenburg, Sweden.

^2^ University of Gothenburg Centre for Person-Centred Care (GPCC), Sahlgrenska Academy, Box 457, 405 30, University of Gothenburg, Sweden.

^3^ Institute of Neuroscience and Physiology, Department of Health and Rehabilitation, Sahlgrenska Academy, University of Gothenburg, Box 455, SE-40530 Gothenburg, Sweden.

^4^ Palliative Centre, Sahlgrenska University Hospital, Box 30110, 400 43, Gothenburg, Sweden.

^5^ Cavernöst Angiom Sverige (CASE), 18645 Vallentuna, Sweden.

^6^ Department of Computer Science and Media Technology, DVMT, 211 19, Malmö University, Malmö, Sweden.

^7^ Department of Applied Information Technology, University of Gothenburg, Box 100, 41296, Gothenburg, Sweden.

Table of Contents

[Supplementary file 1. Study Protocol 3](#_Toc182315196)

[Supplementary file 2. List of the 65 Piloted Outcomes 12](#_Toc182315197)

[Table S1. Outcomes and help text / Swedish to English Translations 15](#_Toc182315198)

[Figure S1. Consort Flow Chart 21](#_Toc182315199)

[Table S2. Round One Scoring Results 22](#_Toc182315200)

[Table S3. Round Two Scoring Results (All Outcomes) 26](#_Toc182315201)

[Table S4. Rating Changes Between Round One and Round Two 32](#_Toc182315202)

# **Supplementary file 1. Study Protocol**

Core Outcomes for the Evaluation of Healthcare Programmes – An Expert Panel Study

Introduction

During the past decade Sweden has experienced an exponential rise in healthcare costs of approximately 32%, totalling 11% of the country’s gross domestic product (1). Despite this substantial outlay, the healthcare landscape in Sweden is plagued with inefficiencies, such as long waiting times, socio-economic health inequalities and ineffective healthcare programmes (2). Person-centred care (PCC) is being advocated as a new, more effective care programme that helps to combat the current strain on resources, whilst maintaining high levels of care. Person-centred care is an approach based on ethical principles, whereby a patient is regarded as an equal ‘partner’ in the design and implementation of their own care (2). Several studies that have evaluated the benefits of PCC have highlighted the effectiveness of this care programme by demonstrating patient improvements in outcomes such as self-efficacy, empowerment, disease management, clinical outcomes, physical functioning and health-related quality of life (3–5).

Aligning interventions with PCC can improve the coordination and access to healthcare services, however, within a healthcare system funded by taxes, production needs to be guided by clear principles for prioritising between the alternative use of resources (3). The Swedish model for prioritising comprises of an ethical platform for priority-setting in healthcare, including cost-effectiveness (1). Within cost-effectiveness evaluations resources are allocated based on the cost of gaining one additional unit of some health effect, such as a quality adjusted life year (QALY), combining health-related quality of life with survival data (6). What cost-effectiveness fails to account for are the aforementioned outcomes where PCC has been proven effective, and although decision-makers and healthcare professionals acknowledge the significant improvement PCC has on patient outcomes, there is no consensus in how to measure and interpret the results of PCC when prioritising resources (2).

Person-centred, ehealth interventions are a further development in the field of PCC, combining the benefits of PCC, whilst presenting care programmes that emphasis cost-saving and efficiency using technological modalities (6). However, the lack of strong empirical evidence for ehealth creates barriers for investment and development, limiting its integration into mainstream healthcare. The inability to make concrete conclusions about ehealth outcomes means that cost-effectiveness studies are either lacking information or are simply not comparable (6). This means that although impactful, many outcomes that are essential to person-centred, ehealth interventions are misconstrued and do not accurately reflect the benefits these care programmes provide. To aid decision-makers and researchers when evaluating person-centred, ehealth interventions, there requires uniformity and transparency through the consistent use and reporting of core outcomes (5).

A core outcome set (COS) is an agreed minimum set of outcomes that should be measured and reported in all clinical trials of a specific disease or population group, however, for PCC challenges remain in defining what outcome measures are most suitable to evaluate (7). A review (8) that analysed 27 PCC interventional studies found that 163 separate outcome measures were used. To effectively evaluate and compare the benefits of PCC, there needs to be consensus both nationally and internationally on the core outcomes that should be measured in clinical trials. This lack of transparency means that decision-makers are unable to evaluate the true cost-effectiveness of PCC, potentially reducing the likelihood that these interventions are adopted. Through the inclusion of PCC outcomes in cost-effectiveness analyses, a more robust representation of PCC will inform decision-making, which, based on previous research, should encourage a greater adoption of PCC interventions in healthcare (2,9,10).

Purpose & Aims

The purpose of this project is the development of a core outcome set for the evaluation of healthcare programmes, such as person-centred care and eHealth. This will allow for better informed decision- making from all involved stakeholder groups in the design, implementation, and evaluation of healthcare programmes.

Methods

In keeping with the recommendations outlaid by the Core Outcome Measures in Effectiveness Trials (COMET) initiative, an expert panel study, using the Delphi method, will be conducted. The study will consist of a pilot study, 2 to 3 sequential questionnaires rounds and a consensus meeting. The protocol has been designed in keeping with the Core Outcome Set STAndards for Development: The COS-STAD recommendations (11).

Expert Panel Study

A Delphi study is defined by its use of subject matter ‘experts’ that provide information on group opinion by way of a series of questionnaire rounds with controlled feedback mechanisms (12). The methodology is considered advantageous because it ensures participant anonymity, avoiding the effect of dominant individuals, and can be circulated to large numbers with wide geographic dispersion (13). When establishing a ‘Core Outcome Set’, the Delphi method is often used to achieve consensus of opinions from experts on the importance of different outcomes in sequential questionnaire rounds. Responses from each question are collated based on stakeholder groups, and later presented to the participants during the subsequent rounds. Providing participants with the opportunity to review their own scores compared against the scores of other stakeholder groups allows them to consider the views of others before re-scoring the questions, possibly influencing a change in their initial responses. With no direct communication between participants this feedback method provides a mechanism for reconciling different opinions of stakeholders and is therefore, critical to achieving consensus (11).

Pilot Study

To compliment the outcomes retrieved through systematic literature analysis, a pilot study will be conducted that includes 2 representatives from each of the stakeholder groups: Decision-makers, researchers, healthcare providers and patients. The pilot, or pretesting work, will be conducted using cognitive or ‘think aloud’ interviews, whereby the stakeholders are asked to read, reflect, and provide feedback on the questionnaire categories and questions. As experts within their respective stakeholder group the participants will also be asked to suggest outcomes that are not present within the pilot questionnaire. Suggested outcomes will be reviewed and if suitable, added to the final questionnaire. The stakeholder group representatives in the pilot study will also form the ‘Study Management Group’, that will take part in a consensus meeting at the conclusion of the study.

**The pilot study has two main aims:**

1. To identify, within the questionnaire, areas for clarity or where the language used is not appropriate or understandable.
2. To identify outcomes that are not present in the initial pilot questionnaire, that may be added to first round of the expert panel study.

Participants

Decisions regarding the appropriate number of participants to include in a Delphi study are made pragmatically and are not based on statistical power (7,13–15). Most importantly, the type, and total number of selected participants must provide an overarching representation of the study’s aims. A minimum of 15 participants will be recruited within each of the 4 stakeholder groups, ensuring that the selected participants are experts, with a deep understanding of the issues within their respective group. The study will look to recruit beyond the 15participant minimum as the greater the number of participants representing each stakeholder group, the better, both in terms of the ‘Core Outcome Set’ being generalisable to future patients and in convincing other stakeholders of its value (7).

Stakeholder Groups

***Decision-makers***
Decision-makers within this study are defined as those people responsible for deciding on the implementation of healthcare interventions. This includes politicians, government representatives and non-patient facing hospital managers.

***Researchers***
This group will include academic representatives that, with evidence-based research, inform decision-makers.

***Healthcare Providers***
This group will include patient-facing healthcare providers, doctors, nurses, physiotherapists, occupational therapists etc.

***Patients / Patient Representatives***
This group represents the users of healthcare interventions and includes patients/people who have been affected by a chronic illness/disease for longer than 3 months and patient groups representatives.

Sampling

Purposive sampling, whereby the intentional selection of experts is based on their ability to elucidate a specific theme, concept, or phenomenon is most prominent within Delphi studies (11,16). Purposive sampling will be used to select the ‘Study Management Group’, the experts representing each of the respective stakeholder groups. Further recruitment for the study will adopt two different sampling methods. Alongside continued purposive sampling, snowball sampling will also be adopted. Members of the ‘study management group’ will be asked at the conclusion of the pilot study to recommend participants they believe are suitable to take part in the study.

Data Collection

Participants that consent to participating in the study will be provided access to DelphiManager, a bespoke software designed by the University of Liverpool specifically for conducting expert panel studies. Round 1 of the expert panel study will commence in early to mid-October, with proceeding rounds running through until the end of the year. Although participants will be aware of the stakeholder group they belong to, their identities will remain anonymous to one another throughout the data collection and analysis process. Data collected within DelphiManager are held securely on a University of Liverpool server and complies with GDPR regulations. Data extracted from DelphiManager by the research team will be stored electronically in a secure and password protected computer, accessible only by the head researcher.

Rounds

According to the Core Outcome Measures in Effectiveness Trails (COMET), (7) Delphi studies should have a minimum of 2 rounds, with 3 rounds being the most common. The number of rounds can have a direct effect on the attrition rates of participants (15). Therefore, a more pragmatic approach is to allow for saturation or consensus to determine the number of rounds required (17). The project will consist of 2-3 scoring rounds and a final round or “consensus meeting”:

**Round 1:** Alongside demographic questions, participants will score outcomes on the questionnaire from 1 to 9 (9-point Likert scale), with 9 being of greatest importance and 1 of least importance (more details on scoring are discussed later in this protocol). The questions are formulated from outcomes identified in a systematic review and outcomes suggested by the ‘study management group’ during the pilot study. The questionnaire will also include open-ended questions where participants can suggest outcomes that were not included in the questionnaire for possible inclusion in round 2.

**Round 2:** In round 2, participants will re-score the outcomes whilst having the opportunity to view the combined round 1 scores of their own, and the other stakeholder groups. If a participant chooses to change their previous rounds score, a box will be provided where they will have the opportunity to explain what motivated their decision. At the end of round 2, outcomes that have been scored 7 to 9 by at least 70% of the participants and 1 to 3 by no more than 15% are deemed to have reached consensus based on the predetermined consensus criteria. The movement of outcomes in and out of the consensus criteria will form the basis for determining if the study has achieved saturation. Significant changes in the scores of outcomes between round 1 and round 2 signify that there is no saturation, and the outcomes will be scored again in the 3rd round.

**Round 3:** Round 3 will follow the same protocol as round 2, and participants will re-score outcomes whilst viewing the results from the 2nd round.

**Final Round (Consensus Meeting):** The study management group will participate in a consensus meeting to discuss the outcomes that have met the consensus criteria and are therefore proposed to be included in the final core outcome set. The outcomes that have not achieve consensus, will also to be discussed to ensure that their exclusion from the core outcome set is appropriate. Nominal group technique has been recommended for this approach as it provides a more open forum for individual opinions, regarded as essential when consensus between groups of varying expertise is required (18).

Table 1. Overview of the Study Rounds & Consensus Meeting

| **Rounds** | **Key criteria:** |
| --- | --- |
| Round 1 | - Demographic questions - Scoring solely from one’s own perspective - Open-ended questions at the end of the questionnaire to suggest further outcomes |
| Round 2 | - Suggested outcomes from round 1 will be added for scoring in round 2 - Criteria for consensus: Score of 7-9 by at least 70% of participants with no more than 15% scoring 1-3 |
| Round 3 | - Round 3 will be omitted if the results of the second round suggest consensus is already achieved / saturation of results - Round 3 will follow the same criteria as round 2 |
| Round 4: Consensus Meeting | - Consensus meeting: Study Management Group - Nominal Group Technique Method |

Once the questionnaire is sent out, each respective round will remain open for 2-4 weeks but can be kept open longer if the response rates are low. Participants will receive weekly reminders through DelphiManager if they have not yet responded to the questionnaire. At the end of each round approximately 1-2 weeks are required to analyse the data and prepare the results that are then uploaded into DelphiManager for the participants to view in the proceeding round. Due to the extensive and lengthy process of retrieving data through multiple questionnaire rounds, Delphi studies often encounter problems with participant retention (15,17). One of the main challenges is how to best analyse the data of participants that score in the first round but then drop out before re- scoring the subsequent rounds. This is because scores in the first round directly influencing the decisions of participants in the subsequent rounds and therefore these results cannot be dismissed. This study has decided that drop out scores will be recorded and discussed in the final consensus meeting, however, will not be decisive for the final scoring when determining the core outcome set.

Feedback

Reconciliation of different views and perspectives is a key feature in achieving consensus within a Delphi study (7). At the end of rounds 1 and 2 the distribution of scores for each individual outcome are presented based on the results from the stakeholder groups. Previous research (11,19) has highlighted that providing feedback to participants from all stakeholder groups improves consensus between stakeholder groups in terms of reduced variability in responses, and improved agreement on which items to retain at the end of the study. This process allows participants to consider their own score against those of the other stakeholder groups before re-scoring an outcome, with participants being encouraged to explain the reasoning behind changing their score, if they choose to do so.

Scoring

The most common method for scoring is a 9-point Likert scoring system, where outcomes are scored in accordance with their level of importance (7). Most commonly, 1 to 3 signifies an outcome is of *limited importance*, 4 to 6 is *important but not critical*, with 7 to 9 being *critical*. Previous studies (15,17) have highlighted those participants often express difficulty in identifying what differentiates a score of 6 or 7, or a score of 8 instead of 7. Although the most common method, there is a need to clearly define these scores beforehand and provide clear explanations to the participants regarding how they should rationalise their decisions. An ‘unable to score’ category will be included to allow for participants that feel they do no not possess an adequate level of expertise to score individual outcomes.

Consensus

Consensus within Delphi studies is measured based on the agreement or disagreement of an individual participant with a statement, that is later compared with group opinion and the extent to which participants agree or disagree with each other. Although the concept of consensus is fundamental to expert panel studies, what constitutes consensus is not clearly defined within research. It is recommended that regardless of the consensus method used, the criteria should be clearly defined within the protocol (7,11). The method proposed for achieving consensus in this study is based on recommendations from the COMET initiative (7,17) and those most used in research according to systematic reviews on Delphi study methodology (11). Outcomes that have been scored 7 to 9 (*critical)* by 70% or more participants and 1 to 3 (*limited importance)* by no more than 15% are deemed to have reached consensus. This consensus criterion is applied at the end of reach scoring round and determines not just whether consensus has been reached or not, but if there is saturation within the results. The outcomes that achieve consensus, as well as those that do not, will be discussed within the consensus meeting where a final set of ‘core outcomes’ will be developed.

Patient and Public Involvement

Two patient representatives will form an integral part of the research team, actively participating in each step of the project. Patient and public involvement will be designed and reported following the GRIPP 2 (20) checklist for the improved reporting of patient and public involvement in healthcare.

**Steps in Patient and Public Involvement:**

•  Once suitable patient representatives are identified, this will assist in the recruitment of participants for the pilot study and the design of the initial questionnaire.

•  The patient representatives will participate in the pilot study as representatives for the “Patient” stakeholder group, helping to define the outcomes and questionnaire questions.

•  Review the final questionnaire after the pilot.

•  Review the results at the end of the Round 1, participant suggested outcomes and assist with the formulation of new questions that are to be added in round 2.

•  Review the results at the end of round 2 (and at the end of round 3 if consensus is not yet achieved) and be active in decision-making surrounding the outcomes that are to be removed and kept in the final round (Consensus Meeting).

•  Take part in the consensus meeting as representatives of the Patient/Patient Representative stakeholder group.

•  Review of the results at the conclusion of the consensus meeting.

•  Review and provide comments on the data analysis process the final results.

•  Review of manuscripts during the writing process and if applicable, contribute as a writing partner in the final manuscript.

Data Analysis

The data analysis is broken into two parts during a Delphi study, the analysis of results during the rounds and the final results. DelphiManager provides a CSV extract of the results at the end of each round. Bar graphs showing the distribution of scores for each stakeholder group will be presented to the participants when answering the questions in subsequent rounds. Although several statistical methods have been sighted in research, means and frequencies are the most common when calculating and presenting the final questionnaire results for the entire study group (7).

Reporting

Current research has indicated that many trialists, systematic reviewers and guidelines reviewers are not referring to COS studies as a starting point when selecting outcomes in their work (22). Variability in the reporting of COS studies has been highlighted as one of the key factors for the reduced visibility of this type of research. Therefore, the reporting of this Delphi study will follow the guidelines outlined in the Core Outcome Set-STAndards for Reporting: The COS-STAR statement (22).

Self-evaluation

The study will undergo constant evaluation to ensure that the eventual results clearly reflect the answers provided by the stakeholder groups. Each category within the questionnaire will be proceeded by an open question where the participants are able to write if they understood the questions. Instances where the participants have experienced difficulties with a specific category or question will be reviewed by the research group in collaboration with the patient representatives. This process will take place between each round, with adjustments being made before the questionnaire is sent out again. The participants will also be asked to grade the questionnaire in its entirety, with the feedback being considered for subsequent rounds and as a tool for improving future research.

Ethical considerations

The application for ethical approval is concerning the collection of data from those participating in the expert panel study. Selected participants score outcomes based on their own views and opinions, and therefore divulge no personal information about themselves during the study. One question that may pose as sensitive asks the participants if they have “experience of having a long-term / chronic illness lasting longer than 3 months”, however the specific details of the illness are not asked. As the study is developing a core outcome set for healthcare programmes, this question is seen as important to ensure that participants have sufficient knowledge of the healthcare system, based on exposure from having a long-term illness. Each participant’s identity is coded within DelphiManager, with the results being aggregated and presented based on stakeholder groups. Therefore, no personal information can be attached to the participants during the analyse process or within the published results.

References

1. Svensson M. Höga kostnader och låg patientnytta: att värdera insatser i sjukvård. 2022.

2. Gyllensten H, Björkman I, Jakobsson Ung E, Ekman I, Jakobsson S. A national research centre for the evaluation and implementation of person‐centred care: Content from the first interventional studies. Health Expect. 2020 Oct;23(5):1362–75.

3. Sharma T, Bamford M, Dodman D. Person-centred care: an overview of reviews. Contemp Nurse. 2015 Dec;51(2–3):107–20.

4. Ali L, Wallström S, Fors A, Barenfeld E, Fredholm E, Fu M, et al. Effects of Person-Centered Care Using a Digital Platform and Structured Telephone Support for People With Chronic Obstructive Pulmonary Disease and Chronic Heart Failure: Randomized Controlled Trial. J Med Internet Res. 2021 Dec 13;23(12):e26794.

5. Gyllensten H, Koinberg I, Carlström E, Olsson LE, Hansson Olofsson E. Economic evaluation of a person-centred care intervention in head and neck oncology: results from a randomized controlled trial. Support Care Cancer. 2019;27(5):1825–34.

6. Bergmo TS. How to Measure Costs and Benefits of eHealth Interventions: An Overview of Methods and Frameworks. J Med Internet Res. 2015 Nov 9;17(11):e254.

7. Prinsen CAC, Vohra S, Rose MR, King-Jones S, Ishaque S, Bhaloo Z, et al. Core Outcome Measures in Effectiveness Trials (COMET) initiative: protocol for an international Delphi study to achieve consensus on how to select outcome measurement instruments for outcomes included in a ‘core outcome set’. Trials. 2014 Jun 25;15:247.

8. Saarijärvi M, Wallin L, Moons P, Gyllensten H, Bratt EL. Mechanisms of impact and experiences of a person-centred transition programme for adolescents with CHD: the Stepstones project. BMC Health Serv Res. 2021 Dec;21(1):573.

9. Pirhonen L, Bolin K, Olofsson EH, Fors A, Ekman I, Swedberg K, et al. Person-Centred Care in Patients with Acute Coronary Syndrome: Cost-Effectiveness Analysis Alongside a Randomised Controlled Trial. PharmacoEconomics - Open. 2019 Dec;3(4):495–504.

10. Gyllensten H, Haby K, Berg M, Premberg Å. Cost effectiveness of a controlled lifestyle intervention for pregnant women with obesity. BMC Pregnancy Childbirth. Sep;21(1):639.

11. Kirkham JJ, Davis K, Altman DG, Blazeby JM, Clarke M, Tunis S, et al. Core Outcome Set-STAndards for Development: The COS-STAD recommendations. PLoS Med. 2017 Nov 16;14(11):e1002447.

12. Trevelyan EG, Robinson PN. Delphi methodology in health research: how to do it? Eur J Integr Med. 2015 Aug 1;7(4):423–8.

13. de Villiers MR, de Villiers PJT, Kent AP. The Delphi technique in health sciences education research. Med Teach. 2005 Nov;27(7):639–43.

14. How to train your oracle: The Delphi method and its turbulent youth in operations research and the policy sciences [Internet]. [cited 2022 May 11]. Available from: http://journals.sagepub.com/doi/epub/10.1177/0306312718798497

15. Biggane AM, Williamson PR, Ravaud P, Young B. Participating in core outcome set development via Delphi surveys: qualitative interviews provide pointers to inform guidance. BMJ Open. 2019 Nov;9(11):e032338.

16. Robinson RS. Purposive Sampling. In: Michalos AC, editor. Encyclopedia of Quality of Life and Well-Being Research [Internet]. Dordrecht: Springer Netherlands; 2014 [cited 2022 May 11]. p. 5243–5. Available from: https://doi.org/10.1007/978-94-007-0753-5_2337

17. Barrington H, Young B, Williamson PR. Patient participation in Delphi surveys to develop core outcome sets: systematic review. BMJ Open. 2021 Sep;11(9):e051066.

18. McMillan SS, King M, Tully MP. How to use the nominal group and Delphi techniques. Int J Clin Pharm. 2016 Jun;38(3):655–62.

19. Meyer DD, Kottner J, Beele H, Schmitt J, Lange T, Hecke AV, et al. Delphi procedure in core outcome set development: rating scale and consensus criteria determined outcome selection. J Clin Epidemiol. 2019 Jul 1;111:23–31.

20. Staniszewska S, Brett J, Simera I, Seers K, Mockford C, Goodlad S, et al. GRIPP2 reporting checklists: tools to improve reporting of patient and public involvement in research. BMJ. 2017 Aug 2;358:j3453.

21. Thompson L, Hill M, Lecky F, Shaw G. Defining major trauma: a Delphi study. Scand J Trauma Resusc Emerg Med. 2021 May 10;29(1):63.

22. Kirkham JJ, Gorst S, Altman DG, Blazeby JM, Clarke M, Devane D, et al. Core Outcome Set-STAndards for Reporting: The COS-STAR Statement. PLoS Med. 2016 Oct;13(10):e1002148.

# **Supplementary file 2. List of the 65 Piloted Outcomes**

1. Effect on functional movement
2. Effect on physical activity
3. Effect on the ability to perform household activities
4. Effect on the ability to perform personal activities in daily life
5. Effect on the ability to perform job activities
6. Effect on the ability to participate in social activities
7. Effect on pain
8. Effect on weakness
9. Effect on fatigue
10. Effect on sleep disturbances
11. Effect on appetite
12. Effect on dizziness
13. Effect on depression
14. Effect on whether the patient feels immobile and tense
15. Effect on catastrophic thoughts/catastrophizing
16. Effect on access to resources for more severe illness/health issues/symptoms
17. Effect on access to resources tailored to the type of disease
18. Effect on access to resources for individuals with life-limiting conditions and at the end of life
19. Effect on access to resources for individuals with rare diseases
20. Effect on access to resources for individuals with diseases where only one intervention is available
21. Effect on access to resources tailored to the needs of different population groups
22. Effect on access to all types of healthcare
23. Effect on access to healthcare that meets the needs of the population
24. Effect on accessibility in healthcare
25. Effect on healthcare costs so individuals do not experience financial burdens due to needing care
26. Effect on conditions for adapting/tailoring healthcare based on population needs
27. Effect on whether healthcare can be adapted to individual needs
28. Effect on whether knowledge of a person's life history can be used in care planning
29. Effect on whether individual needs can be addressed at each care meeting
30. Effect on conditions for allocating sufficient time for each caregiver to meet the patient's needs
31. Effect on financial situation allowing an enjoyable lifestyle
32. Effect on the opportunity to form an opinion on smaller and larger political issues, express them, and feel respected
33. Effect on the opportunity to acquire the education and experience needed to work with or engage in interesting and meaningful activities
34. Effect on the opportunity to live in a pleasant environment
35. Effect on the opportunity to have a satisfying profession or other engaging activities
36. Ability to feel safe and not be afraid of burglary, vandalism, or other forms of threat or violence
37. Effect on the opportunity to have affordable and stable/permanent housing perceived as functional and well-suited to one's needs
38. Effect on problem-solving skills
39. Effect on the ability to set and achieve goals
40. Effect on the ability to use effective coping strategies in setbacks
41. Effect on the ability to do what makes one feel valuable
42. Effect on the ability to think about the future without worries
43. Effect on conditions for receiving love, friendship, and support
44. Effect on conditions for feeling joy and pleasure
45. Effect on total medication costs
46. Effect on the patient's own cost for medication
47. Effect on total healthcare costs
48. Effect on patient fees for healthcare
49. Effect on society's total costs of ill health, including care, assistance, production
50. Effect on the patient's total costs of ill health, including care, assistance, and loss of income
51. Effect on how much time the patient needs to spend on care or self-care due to ill health
52. Effect on the extent to which the patient's income is affected by ill health
53. Effect on the extent to which the patient's leisure time is affected by ill health
54. Effect on how much time relatives/closest family members need to spend on caring for the patient's condition
55. Effect on the extent to which relatives/closest family members' income is affected by the patient's ill health
56. Effect on travel expenses to/from healthcare meetings
57. Effect on time spent traveling to/from healthcare meetings
58. Evaluation time horizon
    1. 1 week
    2. 4 weeks
    3. 3 months
    4. 1 year
    5. 3 years
    6. 5 years
    7. 10 years
    8. Lifelong evaluation

# **Table S1. Outcomes and help text / Swedish to English Translations**

| **Swedish Language Outcomes and Text** | | **Research Team Translation to English** | |
| --- | --- | --- | --- |
| **Utfall (Swedish)** | **Hjälptext (Swedish)** | **Outcome (English)** | **Help text English** |
| Funktionella rörelser | (Böja sig, knäsittande, gående, sittande, stående) | Functional movements | (Bending, kneeling, walking, sitting, standing) |
| Fysisk aktivitet | (Inaktiv, lätt träning, medelhård/intensiv träning, hård/intensiv träning) | Physical activity | (Inactive, light exercise, moderate/intense exercise, vigorous/intensive exercise) |
| Förmåga att utföra hushållsaktiviteter | (Matlagning, tvätt, städning) | Ability to perform household activities | (Cooking, laundry, cleaning) |
| Förmåga att utföra personliga aktiviteter i dagliga livet | (Att kunna sköta sin hygien, ta på sig kläder etc.) | Ability to perform personal activities in daily life | (To be able to manage one's hygiene, put on clothes, etc.) |
| Förmåga att utföra arbete och studier | (Genomföra arbetsuppgifter och studera) | Ability to work and study | (Complete work tasks and studying) |
| Förmåga att delta i sociala aktiviteter | (Utföra aktiviteter tillsammans med andra) | Ability to participate in social activities | (Engaging in activities with others) |
| Smärta / värk | (Hur mycket smärta / värk man känner i kroppen) | Pain / Discomfort | How much pain / discomfort one feels in the body) |
| Ångest och / eller depression | (Hur mycket ångest och / eller hur deprimerad man känner sig) | Anxiety and / or Depression | (How much anxiety and/or how depressed one feels) |
| Patientens livskvalitet | (Livskvalitet som ett brett och sammansatt mått på patientens egen värdering av sitt fysiska, psykiska och sociala välbefinnande) | Patient's quality of life | (Quality of life as a broad and comprehensive measure of the patient's own assessment of their physical, mental, and social well-being) |
| Anhörigas / närståendes livskvalitet | (Livskvalitet som ett brett och sammansatt mått på anhörigas / närståendes egen värdering av sitt fysiska, psykiska och sociala välbefinnande) | Significant others' quality of life | (Quality of life as a broad and comprehensive measure of significant others’ own assessment of their physical, mental, and social well-being) |
| Lidande | Lindring vid ohälsa som begränsar individens tillgång till sitt liv, även då det inte går att bota. | Suffering | (Relief from illness that limits an individual's access to their life, even when it cannot be cured) |
| Förmåga att kunna känna trygghet och säkerhet | (Olika aspekter av livet, såsom att inte behöva oroa sig för våld, för sin ekonomi, för oönskade sociala förändringar) | Ability to feel safe and secure | (Various aspects of life, such as not worrying about violence, finances, or unwanted social changes) |
| Förmåga att kunna vara oberoende av andra | (Olika aspekter av livet, såsom att känna att man inte är beroende av andra (anhöriga, vården) utan att man kan klarar sig själv) | Ability to be independent of others | (Various aspects of life, such as feeling that one is not dependent on others (family, healthcare) and can manage on their own). |
| Förmåga att kunna göra det som får en att känna sig värdefull | (Att kunna utföra aktiviteter som får en att känna sig värdefull i olika aspekter av livet) | Ability to do what makes one feel valuable | (Being able to perform activities that make one feel valuable in different aspects of life) |
| Förmåga att kunna uppleva sitt liv som meningsfullt | (Att kunna känna att ens existens har syfte och signifikans) | Ability to experience one's life as meaningful | (Being able to feel that one's existence has purpose and significance) |
| Förmåga att hitta lösningar på problem som uppstår | (Att kunna flexibelt använda problemlösningsförmåga för att hantera problem i olika aspekter av livet) | Ability to find solutions to problems that arise | (Being able to flexibly problem-solving skills to manage challenges in various aspects of life) |
| Förmåga att kunna sätta upp och nå mål | (Att kunna sätta upp både små och stora mål och sedan klara av dem) | Ability to set and reach goals | (Being able to set both small and large goals and then reach them) |
| Förmåga att kunna nå resultat och framgångar | (Att kunna uppnå mål och överträffa sina eller andras förväntningar) | Ability to achieve results and successes | (Being able to reach goals and exceed one's own or others' expectations) |
| Förmåga att kunna tänka på framtiden utan bekymmer | (Att kunna tänka framåt utan att känna oro och ångest i olika aspekter av livet) | Ability to think about the future without concern | (Being able to look ahead without feeling anxiety and concern in different aspects of life) |
| Förmåga att kunna använda effektiva coping-strategier vid motgångar | (Att kunna använda adaptiva och anpassade strategier för att hantera att någonting inte gick som man ville) | Ability to effectively use coping strategies when faced with adversity | (Being able to use adaptive and tailored strategies to handle situations where things didn’t go as planned) |
| Förmåga att kunna återhämta sig från motgångar eller trauma | (Att kunna växa och utveckla sig som person, hantera sina reaktioner samt tänka kreativt och positivt även vid svåra situationer) | Ability to recover from setbacks or trauma | (Being able to grow and develop as a person, manage one's reactions, and think creatively and positively even in difficult situations) |
| Förutsättningar att få/känna kärlek, vänskap och stöd | (Att kunna ingå i och få ut någonting av nära sociala relationer) | Ability to experience love, friendship, and support | (Engaging in and deriving satisfaction from close social relationships) |
| Förutsättningar att känna glädje och nöje | (Förmågan att glädjas av små och stora saker i olika aspekter av livet) | Ability to feel happiness and satisfaction | (The ability to find joy in small and big things in various aspects of life) |
| Känsla att bli sedd som en person som är kapabel men också sårbar | Patientens upplevelse att bli sedd som en person som har styrkor men också kan uppleva utmaningar och hälsohinder på samma gång. | Feeling of being seen as a person who is both capable and vulnerable | (The patient's experience of being seen as an individual with strengths but also experiences health challenges and obstacles simultaneously) |
| Förutsättningar att planera vården utifrån patientens livshistoria och villkor | (Om patientens unika erfarenheter, förutsättningar, tillgångar och problem kan integreras i behandlingen) | Preconditions to plan care based on the patient's lived experience and circumstances | (Whether the patient's unique experiences, conditions, assets, and problems can be integrated into the treatment) |
| Förutsättningar att anpassa planeringen till patientens behov | (Hur väl det går att justera vården specifikt utefter patientens egna behov, till exempel frekvensen av vårdmöten) | Preconditions to adapt planning based on the patient's needs | (How well healthcare can be adjusted according to the patient's specific needs, such as the frequency of healthcare meetings) |
| Förutsättningar att anpassa vården utifrån mål uppsätta tillsammans med patienten | (Förutsättningar till flexibilitet och lyhördhet i vårdmöten för att nå gemensamma mål) | Ability to adapt care based on goals set together with the patient | (Ability to flexible and responsive in healthcare meetings to achieve shared goals) |
| Förutsättningar att patienten får sina frågor och oro bemött | (Förutsättningar att kontinuerligt kunna få stöd och diskutera sina frågor) | Conditions for the patient to have their questions and concerns addressed | (Conditions for continuously receiving support and discussing questions) |
| Förutsättningar att anhöriga / närstående får sina frågor och oro bemött | (Förutsättningar att kontinuerligt kunna få stöd och diskutera sina frågor) | Conditions for significant others to have their questions and concerns addressed | (Conditions for continuously receiving support and discussing questions) |
| Förutsättningar att uppmärksamma förändringar i patientens behov | (Om det finns tid och en naturlig möjlighet för att uppmärksamma att patientens hälsa kan ha förändrats sedan vården inleddes) | Preconditions for noticing changes in the patient's needs | (Whether there is time and a natural opportunity to notice that the patient's health may have changed since the start of care) |
| Förutsättningar i vården bidrar till att patienten har kontroll över den fortsatta vårdprocessen | (Patienten vet vilka egenvårdsinsatser och planerade vårdinsatser som ska ske och har förutsättningar att genomföra dessa) | Conditions of healthcare contributing to the patient having control over the continued care process | (The patient knows which self-care actions and planned care interventions that should take place and has the conditions to carry them out) |
| Patientupplevelse | (Patientens upplevelse av vården, har fått svar på sina frågor, bemötande, patientnöjdhet) | Patient experience | (The patient's experience of healthcare, getting answers to their questions, treatment, patient satisfaction) |
| Vårdpersonalens resursfördelning | (Hur flexibel vården är i att tilldela extra resurser (tid, pengar, expertis) där det behövs som mest, utifrån olika patienters behov) | Health worker’s resource allocation | (How flexible healthcare is in allocating extra resources (time, money, expertise) where needed most, based on different patients' needs) |
| Vårdpersonalens arbetsbörda | (Om det bidrar till att anställda i vården får mer eller mindre att göra, tidspress) | Health worker’s workload | (Whether it contributes to health workers having more, or less, to do, time pressure) |
| Vårdpersonalens arbetsmiljö | (Hur den sociala, fysiska och organisatoriska miljön på vårdpersonalens arbetsplats förändras, inklusive resurser, miljö) | Health worker's work environment | (How the social, physical, and organizational environment in the workplace changes, including resources, and the environment) |
| Vårdpersonalens etiska stress | (Hur moraliskt korrekt vårdpersonalen tycker att det är att ge vården, och hur det påverkar deras samvetskänslor att utföra den) | Health worker's ethical stress | (How morally correct health workers feel about providing care, and how it affects their conscience to perform it) |
| Tillgång till hälso- och sjukvård som motsvarar alla behov | (Huruvida det överhuvudtaget finns ett utbud av vård som tillgodoser patienters behov, inklusive patienter med särskilda behov såsom sällsynta eller allvarliga sjukdomar) | Access to healthcare that meets all needs | (Whether there is an offering of healthcare that addresses patients' needs, including patients with special needs such as rare or serious illnesses) |
| Tillgänglighet till olika typer av hälso- och sjukvård | (Huruvida den vård som finns är åtkomlig: inte för dyr, finns nära patienten, inte för långa väntetider, tillgänglig för personer med funktionshinder) | Accessibility to different types of healthcare | (Whether the available care is accessible: not too expensive, near to the patient, with reasonable wait times, and accessible to people with disabilities) |
| Patienten upplever att hen bli bemött med respekt som en jämlike | Att patienten känner sig bemött som en person med lika värde oavsett bakgrund, kön, ålder eller andra faktorer. | The patient feels they are treated with respect as an equal | (The patient feels treated as a person of equal worth, regardless of background, gender, age, or other factors) |
| Möjlighet att kunna välja vårdkontakt | Att patienten har möjlighet att välja den vårdkontakt som de känner sig mest bekväm med eller som passar bäst för deras behov. | Possibility to choose a healthcare contact | (The patient has the opportunity to choose the care provider they feel most comfortable with or that best meets their needs) |
| Patientens och vid behov, närstående/anhörigas perspektiv beaktas i alla beslut om kommande vård | Att både patienten och vid behov, deras närstående/anhörigas åsikter och behov vägs in vid beslut om vård. | The patient's perspective and, if required, significant others' perspectives, are taken into account in all decisions regarding future care. | (Considering both the patient's and, if necessary, significant others’ opinions and needs in decisions about care) |
| Patienten och vid behov, närstående/anhöriga är förberedda på vad som skall ske | Att hälso- och sjukvårdspersonal säkerställer att patient och vid behov, deras närstående/anhörigas är införstådda och förbereda på vad som kan hända på kort och lång sikt. | The patient and, when necessary, significant others', are prepared for what will happen | (Health workers ensure that the patient and, if needed, significant others, are informed and prepared for what may happen in the short and long term) |
| Vårdens förmåga att samverka externt | Att kunna samarbeta och kommunicera externt, med andra aktörer och organisationer inom hälso- och sjukvården. | Healthcare's ability to collaborate externally | (The ability to collaborate and communicate externally, with other actors and organizations in healthcare) |
| Vårdens förmåga att samverka internt | Att kunna samarbeta och kommunicera internt, inom organisationen. | Healthcare's ability to collaborate internally | (The ability to collaborate and communicate internally, within the organization) |
| Kontinuitet i vården | Att kunna ge en sammanhängande och samordnad vård till patienten över tid. | Continuity in healthcare | (The ability to provide connected and coordinated care to the patient over time) |
| Förmåga att se den unika personen | Att kunna se helheten och hela personen i sitt sammanhang. | Ability to see the unique individual | (Being able to see the entirety and whole person in their context) |
| Vårdpersonalens förmåga att arbeta självständigt utifrån sin kompetens | Hälso- och sjukvårdspersonalens förmåga genomföra arbetssätt baserat på sina kunskaper och färdigheter. Detta innebär att de kan fatta kliniska beslut och utföra uppgifter inom sitt ansvarsområde. | Health worker’s ability to work independently based on their competence | (The ability of health workers to carry out practices based on their knowledge and skills. This means they can make clinical decisions and perform tasks within their area of responsibility) |
| Patientens utgifter för sjukvård och läkemedel | (Hur mycket det kostar för patienten) | The patient's expenses for healthcare and medication | (How much it costs for the patient) |
| Samhällets kostnader för sjukvård | (Hur mycket det kostar för samhället (i första hand regionerna) som helhet att ge vården) | Society's costs for healthcare | (How much it costs society, (primarily the regions), as a whole to provide healthcare) |
| Samhällets kostnader för läkemedel | (Hur mycket det kostar för samhället (regionerna och staten) som helhet att ge vården) | Society's costs for medications | (How much it costs society, (regions and the state), as a whole to provide healthcare) |
| Patientens/brukarens utgifter för kommunal omsorg (särskild boende, hemtjänst) | (Hur mycket det kostar för brukare) | The patient's/service user's expenses for municipal care (nursing homes, home help services) | (How much it costs for the patient/service user) |
| Samhällets kostnader för kommunal omsorg (särskild boende, hemtjänst) | (Hur mycket det kostar för samhället (i första hand kommunerna) att ge olika former av omsorgsstöd) | Society's costs for municipal care (nursing homes and home help services) | (How much it costs society (primarily municipalities) to provide various forms of care support) |
| Påverkan på patientens arbetstid eller inkomst av ohälsan | (Hur mycket tid som patienten inte kan jobba för att de behöver ta hand om ohälsan, och de ekonomiska konsekvenserna av detta) | The impact of illness on the patient's working hours or income | (How much time the patient cannot work because they need to manage their illness, and/or the financial consequences of this) |
| Påverkan på anhörigas / närståendes arbetstid eller inkomst av patientens ohälsa | (Hur mycket tid som anhöriga / närstående inte kan jobba för att de behöver ta hand om patientens ohälsa, och de ekonomiska konsekvenserna av detta) | The impact on significant others’ working hours or income due to the patient's illness | (How much time significant others cannot work because they need to care for the patient's illness, and/or the financial consequences of this) |
| Fritid som patienten lägger på vård eller egenvård | (Tidsåtgång för hantering av ohälsan, till exempel för medicinering, hanterande av symtom) | Leisure time the patient allocates to care or self-care | (Time spent handling illness, for example managing medication and symptoms) |
| Fritid som anhöriga / närstående lägger på patientens vård eller egenvård | (Tidsåtgång för hantering av ohälsan, till exempel för medicinering, hanterande av symtom) | Leisure time significant others allocate to patient care or self-care | (Time spent handling illness, for example managing medication and symptoms) |
| Tidsåtgång och resekostnader till / från vård | (Kostnader och tidsåtgång relaterade till kollektivtrafik, bilkörning) | Time spent and travel costs to/from healthcare | (Costs and time related to public transportation, driving) |
| Resursanvändning för patientnära arbete | Tidsåtgång för patientnära arbete jämfört med nuvarande arbetssätt. | Resource use for patient-facing work | (Time spent on patient-facing tasks compared to current ways of working) |
| Resursanvändning för administration | Tidsåtgång för administration jämfört med nuvarande arbetssätt. | Resource use for administration | (Time spent on administration compared to current work methods) |
| Tillgång till hälsoinformation | (Om informationen uppfattas som tillgänglig snabbt och intuitivt för patienter och eller anhöriga/närstående samt vårdpersonal, eller om man måste leta efter den) | Access to health information | (Whether the information is perceived as quickly and intuitively accessible for patients and/or significant others, as well as health workers, or if one must search for it) |
| Tillgänglig hälsoinformation | (Om informationen finns anpassad för patientens nivå av teknologisk förståelse, funktionsnedsättning eller förståndshandikapp) | Accessible health information | (Whether the information is adapted to the patient's level of technological understanding, physical or intellectual disability) |
| Upplevelse av säkerhet i hantering av personuppgifter | (Att uppfatta att ingen ovederbörlig person har tillgång till ens privata uppgifter) | The experience of safety in the handling of personal information | (Perceiving that no unauthorized person has access to one's private information) |
| Upplevelse av meningsfullhet i tjänster och funktioner | (Känsla av att de e-hälsorelaterade tjänster och funktioner som finns faktiskt bidrar till att hjälpa deras hälsa) | Experiencing services and functions as meaningful | (Feeling that the e-health-related services and functions actually contribute to improving health) |
| Upplevelse av systemets pålitlighet | (Att systemen som används är stabila och inte kraschar eller strular när de ska användas) | Experiencing the system as reliable | (Whether the systems used are stable and do not crash or malfunction when they are being used) |

Recruitment emails n = 129

Baseline

Withdrew n = 0

Incomplete n = 6

**Completed n = 6**

Withdrew n = 0

Incomplete n = 1

**Completed n = 16**

Withdrew n = 0

Incomplete n = 1

**Completed n = 15**

Withdrew n = 1

Incomplete n = 3

**Completed n = 9**

Withdrew n = 1

Incomplete n = 4

**Completed n = 13**

Withdrew n = 0

Incomplete n = 0

**Completed n = 16**

Withdrew n = 0

Incomplete n = 4

**Completed n = 17**

Withdrew n = 2

Incomplete n = 0

**Completed n = 12**

Managerial decision-maker

n = (14)

Researcher

n = (21)

Health worker

n = (16)

Patient / Patient Representative

n = (18)

Round One

Round Two

**Final analysis (n = 46)**

|  |
| --- |

# **Figure S1. Consort Flow Chart**

# **Table S2. Round One Scoring Results**

**Bold =** Outcomes scored critical by at least one stakeholder group

**Bold** = Outcomes scored critical by all stakeholder groups

| **Outcome** | Scoring Category | Patient / Patient Representative (%) | Health Worker (%) | Researcher (%) | Managerial Decision-Maker (%) |
| --- | --- | --- | --- | --- | --- |
| Functional movements | Not important | 0 | 20 | 11.8 | 36.4 |
|  | Important | 41.7 | 33.3 | 47.1 | 27.3 |
|  | Critical | 58.3 | 46.7 | 41.2 | 36.4 |
| Physical activity | Not important | 0 | 20 | 5.9 | 18.2 |
|  | Important | 46.2 | 26.7 | 52.9 | 45.5 |
|  | Critical | 53.8 | 53.3 | 41.2 | 36.4 |
| Ability to perform household activities | Not important | 0 | 20 | 5.9 | 16.7 |
|  | Important | 53.8 | 46.7 | 47.1 | 50 |
|  | Critical | 46.2 | 33.3 | 47.1 | 33.3 |
| **Ability to perform personal activities in daily life** | Not important | 0 | 6.7 | 0 | 8.3 |
|  | Important | 0 | 20 | 5.9 | 16.7 |
|  | Critical | 100 | 73.3 | 94.1 | 75 |
| **Ability to work and study** | Not important | 7.7 | 6.2 | 0 | 16.7 |
|  | Important | 7.7 | 31.2 | 11.8 | 50 |
|  | Critical | 84.6 | 62.5 | 88.2 | 33.3 |
| **Ability to participate in social activities** | Not important | 0 | 6.2 | 0 | 8.3 |
|  | Important | 15.4 | 25 | 29.4 | 41.7 |
|  | Critical | 84.6 | 68.8 | 70.6 | 50 |
| **Pain / Discomfort** | Not important | 0 | 6.2 | 0 | 8.3 |
|  | Important | 23.1 | 25 | 29.4 | 16.7 |
|  | Critical | 76.9 | 68.8 | 70.6 | 75 |
| **Anxiety and / or Depression** | Not important | 0 | 6.2 | 0 | 8.3 |
|  | Important | 16.7 | 18.8 | 23.5 | 8.3 |
|  | Critical | 83.3 | 75 | 76.5 | 83.3 |
| **Patient's quality of life** | Not important | 0 | 0 | 0 | 0 |
|  | Important | 7.7 | 0 | 0 | 8.3 |
|  | Critical | 92.3 | 100 | 100 | 91.7 |
| **Significant others' quality of life** | Not important | 0 | 6.2 | 17.6 | 8.3 |
|  | Important | 15.4 | 31.2 | 35.3 | 58.3 |
|  | Critical | 84.6 | 62.5 | 47.1 | 33.3 |
| Capabilities and Support System Outcomes | | | | | |
| **Outcome** | **Scoring Category** | **Patient / Patient Representative (%)** | **Health Worker (%)** | **Researcher (%)** | **Managerial Decision-Maker (%)** |
| **Ability to feel safe and secure** | Not important | 0 | 0 | 0 | 0 |
|  | Important | 7.7 | 12.5 | 17.6 | 36.4 |
|  | Critical | 92.3 | 87.5 | 82.4 | 63.6 |
| Ability to be independent from others | Not important | 0 | 6.2 | 6.2 | 18.2 |
|  | Important | 46.2 | 62.5 | 43.8 | 36.4 |
|  | Critical | 53.8 | 31.2 | 50 | 45.5 |
| **Ability to do those things that makes one feel valuable** | Not important | 0 | 0 | 6.2 | 0 |
|  | Important | 7.7 | 37.5 | 37.5 | 63.6 |
|  | Critical | 92.3 | 62.5 | 56.2 | 36.4 |
| **Ability to experience one's life as meaningful** | Not important | 0 | 0 | 5.9 | 0 |
|  | Important | 0 | 25 | 17.6 | 36.4 |
|  | Critical | 100 | 75 | 76.5 | 63.6 |
| **Ability to find solutions to problems that arise** | Not important | 0 | 0 | 5.9 | 0 |
|  | Important | 23.1 | 25 | 52.9 | 54.5 |
|  | Critical | 76.9 | 75 | 41.2 | 45.5 |
| Ability to set and reach goals | Not important | 0 | 0 | 11.8 | 9.1 |
|  | Important | 30.8 | 31.2 | 52.9 | 45.5 |
|  | Critical | 69.2 | 68.8 | 35.3 | 45.5 |
| Ability to achieve results and success | Not important | 0 | 0 | 29.4 | 9.1 |
|  | Important | 53.8 | 68.8 | 41.2 | 63.6 |
|  | Critical | 46.2 | 31.2 | 29.4 | 27.3 |
| Ability to be able to think about the future without concern | Not important | 0 | 18.8 | 12.5 | 30 |
|  | Important | 38.5 | 50 | 50 | 40 |
|  | Critical | 61.5 | 31.2 | 37.5 | 30 |
| Ability to effectively use coping strategies when faced with adversity | Not important | 8.3 | 0 | 0 | 0 |
|  | Important | 33.3 | 37.5 | 33.3 | 72.7 |
|  | Critical | 58.3 | 62.5 | 66.7 | 27.3 |
| **Ability to be able to recover from adversity or trauma** | Not important | 0 | 0 | 6.2 | 9.1 |
|  | Important | 15.4 | 50 | 37.5 | 63.6 |
|  | Critical | 84.6 | 50 | 56.2 | 27.3 |
| **Ability to experiencing love, friendship, and support** | Not important | 0 | 6.2 | 6.2 | 9.1 |
|  | Important | 7.7 | 31.2 | 31.2 | 36.4 |
|  | Critical | 92.3 | 62.5 | 62.5 | 54.5 |
| **Ability to feel happiness and satisfaction** | Not important | 0 | 0 | 0 | 18.2 |
|  | Important | 15.4 | 43.8 | 43.8 | 27.3 |
|  | Critical | 84.6 | 56.2 | 56.2 | 54.5 |
| Care Process / Organisational Outcomes | | | | | |
| **Outcome** | Scoring Category | Patient / Patient Representative (%) | Health Worker (%) | Researcher (%) | Managerial Decision-Maker (%) |
| **Preconditions to plan care based on the patient's lived experience and needs** | Not important | 0 | 0 | 0 | 0 |
|  | Important | 7.1 | 18.8 | 17.6 | 50 |
|  | Critical | 92.9 | 81.2 | 82.4 | 50 |
| **Preconditions to adapt planning based on the patient's needs** | Not important | 0 | 0 | 0 | 0 |
|  | Important | 0 | 6.2 | 11.8 | 16.7 |
|  | Critical | 100 | 93.8 | 88.2 | 83.3 |
| **Ability to adapt healthcare based on goals set together with the patient** | Not important | 0 | 0 | 0 | 0 |
|  | Important | 0 | 25 | 5.9 | 25 |
|  | Critical | 100 | 75 | 94.1 | 75 |
| **Conditions for the patient to have their questions and concerns addressed** | Not important | 0 | 0 | 0 | 0 |
|  | Important | 7.1 | 18.8 | 17.6 | 16.7 |
|  | Critical | 92.9 | 81.2 | 82.4 | 83.3 |
| **Conditions for significant others to have their questions and concerns addressed** | Not important | 0 | 0 | 0 | 16.7 |
|  | Important | 7.1 | 37.5 | 41.2 | 50 |
|  | Critical | 92.9 | 62.5 | 58.8 | 33.3 |
| **Preconditions for noticing changes in the patient's needs** | Not important | 0 | 0 | 0 | 0 |
|  | Important | 0 | 6.2 | 17.6 | 25 |
|  | Critical | 100 | 93.8 | 82.4 | 75 |
| **Conditions for healthcare to contribute to the patient having control over the ongoing care process** | Not important | 0 | 0 | 0 | 8.3 |
|  | Important | 15.4 | 31.2 | 23.5 | 33.3 |
|  | Critical | 84.6 | 68.8 | 76.5 | 58.3 |
| **Patient experience** | Not important | 7.1 | 0 | 0 | 0 |
|  | Important | 21.4 | 0 | 18.8 | 16.7 |
|  | Critical | 71.4 | 100 | 81.2 | 83.3 |
| Health worker's resource allocation | Not important | 7.7 | 0 | 0 | 0 |
|  | Important | 46.2 | 31.2 | 41.2 | 33.3 |
|  | Critical | 46.2 | 68.8 | 58.8 | 66.7 |
| **Health worker's workload** | Not important | 7.7 | 0 | 0 | 0 |
|  | Important | 38.5 | 25 | 35.3 | 25 |
|  | Critical | 53.8 | 75 | 64.7 | 75 |
| **Health worker's work environment** | Not important | 0 | 0 | 0 | 0 |
|  | Important | 38.5 | 18.8 | 23.5 | 16.7 |
|  | Critical | 61.5 | 81.2 | 76.5 | 83.3 |
| **Health worker's ethical stress** | Not important | 0 | 0 | 0 | 0 |
|  | Important | 38.5 | 18.8 | 23.5 | 25 |
|  | Critical | 61.5 | 81.2 | 76.5 | 75 |
| **Access to healthcare that meets everyone’s needs** | Not important | 7.1 | 6.2 | 0 | 8.3 |
|  | Important | 14.3 | 31.2 | 46.7 | 41.7 |
|  | Critical | 78.6 | 62.5 | 53.3 | 50 |
| **Accessibility to different types of healthcare** | Not important | 7.1 | 18.8 | 11.8 | 9.1 |
|  | Important | 21.4 | 12.5 | 41.2 | 45.5 |
|  | Critical | 71.4 | 68.8 | 47.1 | 45.5 |
| Economic Outcomes | | | | | |
| **Outcome** | Scoring Category | Patient / Patient Representative (%) | Health Worker (%) | Researcher (%) | Managerial Decision-Maker (%) |
| Patient's expenses for healthcare and medication | Not important | 7.7 | 6.2 | 11.8 | 8.3 |
|  | Important | 38.5 | 50 | 58.8 | 58.3 |
|  | Critical | 53.8 | 43.8 | 29.4 | 33.3 |
| **Society's costs for healthcare** | Not important | 0 | 0 | 0 | 0 |
|  | Important | 69.2 | 37.5 | 37.5 | 16.7 |
|  | Critical | 30.8 | 62.5 | 62.5 | 83.3 |
| Society's costs for medications | Not important | 0 | 0 | 0 | 0 |
|  | Important | 61.5 | 50 | 50 | 33.3 |
|  | Critical | 38.5 | 50 | 50 | 66.7 |
| Patient's / service user's expenses municipal care, including nursing homes and home help services | Not important | 8.3 | 6.7 | 12.5 | 9.1 |
|  | Important | 33.3 | 46.7 | 37.5 | 54.5 |
|  | Critical | 58.3 | 46.7 | 50 | 36.4 |
| Society's costs for municipal care, including nursing homes and home help services | Not important | 8.3 | 0 | 0 | 0 |
|  | Important | 58.3 | 46.7 | 43.8 | 36.4 |
|  | Critical | 33.3 | 53.3 | 56.2 | 63.6 |
| The impacts of illness on the patient’s working hours and income | Not important | 0 | 0 | 11.8 | 10 |
|  | Important | 38.5 | 56.2 | 52.9 | 60 |
|  | Critical | 61.5 | 43.8 | 35.3 | 30 |
| The impact on significant others’ working hours or income due to the patient’s illness | Not important | 0 | 0 | 17.6 | 9.1 |
|  | Important | 30.8 | 62.5 | 47.1 | 63.6 |
|  | Critical | 69.2 | 37.5 | 35.3 | 27.3 |
| Leisure time the patient allocates to care or self-care | Not important | 0 | 6.2 | 5.9 | 9.1 |
|  | Important | 53.8 | 68.8 | 76.5 | 72.7 |
|  | Critical | 46.2 | 25 | 17.6 | 18.2 |
| Leisure time the significant other allocates to patient care or self-care | Not important | 0 | 12.5 | 17.6 | 8.3 |
|  | Important | 38.5 | 62.5 | 64.7 | 75 |
|  | Critical | 61.5 | 25 | 17.6 | 16.7 |
| Time spent and travel costs to/from healthcare | Not important | 0 | 18.8 | 11.8 | 16.7 |
|  | Important | 53.8 | 50 | 82.4 | 58.3 |
|  | Critical | 46.2 | 31.2 | 5.9 | 25 |
| Ehealth Outcomes | | | | | |
| **Outcome** | Scoring Category | Patient / Patient Representative (%) | Health Worker (%) | Researcher (%) | Managerial Decision-Maker (%) |
| **Access to health information** | Not important | 7.7 | 0 | 0 | 8.3 |
|  | Important | 15.4 | 50 | 23.5 | 41.7 |
|  | Critical | 76.9 | 50 | 76.5 | 50 |
| **Accessible health information** | Not important | 7.7 | 0 | 0 | 0 |
|  | Important | 23.1 | 50 | 35.3 | 16.7 |
|  | Critical | 69.2 | 50 | 64.7 | 83.3 |
| **The experience of safety in the handling of personal information** | Not important | 0 | 6.2 | 0 | 0 |
|  | Important | 21.4 | 37.5 | 23.5 | 50 |
|  | Critical | 78.6 | 56.2 | 76.5 | 50 |
| **Experiencing services and functions as meaningful** | Not important | 0 | 0 | 0 | 0 |
|  | Important | 7.1 | 25 | 29.4 | 18.2 |
|  | Critical | 92.9 | 75 | 70.6 | 81.8 |
| **Experiencing the system as reliable** | Not important | 0 | 6.2 | 0 | 0 |
|  | Important | 14.3 | 31.2 | 41.2 | 0 |
|  | Critical | 85.7 | 62.5 | 58.8 | 100 |

# Table S3. Round Two Scoring Results (All Outcomes)

Underlined **=** Outcomes included in the final COS

*Cursive* = Outcomes included based on suggestions from round one

| General Health Outcomes | | | | | |
| --- | --- | --- | --- | --- | --- |
| Outcomes | Scoring Category | Patient / Patient Representative (%) | Health Worker (%) | Researcher (%) | Managerial Decision-Maker (%) |
| Functional movements | Not important | 12.5 | 0 | 12.5 | 33.3 |
|  | Important | 37.5 | 50 | 43.8 | 33.3 |
|  | Critical | 50 | 50 | 43.8 | 33.3 |
| Physical activity | Not important | 11.1 | 14.3 | 0 | 16.7 |
|  | Important | 44.4 | 28.6 | 56.2 | 66.7 |
|  | Critical | 44.4 | 57.1 | 43.8 | 16.7 |
| Ability to perform household activities | Not important | 0 | 14.3 | 0 | 16.7 |
|  | Important | 44.4 | 57.1 | 50 | 66.7 |
|  | Critical | 55.6 | 28.6 | 50 | 16.7 |
| Ability to perform personal activities in daily life | Not important | 0 | 0 | 0 | 16.7 |
|  | Important | 0 | 21.4 | 6.2 | 16.7 |
|  | Critical | 100 | 78.6 | 93.8 | 66.7 |
| Ability to work and study | Not important | 11.1 | 6.7 | 0 | 16.7 |
|  | Important | 0 | 40 | 18.8 | 66.7 |
|  | Critical | 88.9 | 53.3 | 81.2 | 16.7 |
| Ability to participate in social activities | Not important | 0 | 0 | 0 | 16.7 |
|  | Important | 11.1 | 26.7 | 12.5 | 33.3 |
|  | Critical | 88.9 | 73.3 | 87.5 | 50 |
| Pain / Discomfort | Not important | 0 | 0 | 0 | 16.7 |
|  | Important | 33.3 | 20 | 25 | 0 |
|  | Critical | 66.7 | 80 | 75 | 83.3 |
| Anxiety and / or Depression | Not important | 0 | 0 | 0 | 0 |
|  | Important | 28.6 | 26.7 | 12.5 | 16.7 |
|  | Critical | 71.4 | 73.3 | 87.5 | 83.3 |
| Patient's quality of life | Not important | 0 | 0 | 0 | 0 |
|  | Important | 0 | 0 | 0 | 0 |
|  | Critical | 100 | 100 | 100 | 100 |
| Significant others' quality of life | Not important | 0 | 6.7 | 12.5 | 16.7 |
|  | Important | 0 | 33.3 | 37.5 | 50 |
|  | Critical | 100 | 60 | 50 | 33.3 |
| *Suffering* | Not important | 0 | 0 | 0 | 0 |
|  | Important | 14.3 | 26.7 | 40 | 50 |
|  | Critical | 85.7 | 73.3 | 60 | 50 |
| Capabilities and Support System Outcomes | | | | | |
| Outcomes | Scoring Category | Patient / Patient Representative (%) | Health Worker (%) | Researcher (%) | Managerial Decision-Maker (%) |
| Ability to feel safe and secure | Not important | 0 | 0 | 0 | 0 |
|  | Important | 11.1 | 6.7 | 18.8 | 33.3 |
|  | Critical | 88.9 | 93.3 | 81.2 | 66.7 |
| Ability to be independent from others | Not important | 0 | 0 | 6.2 | 0 |
|  | Important | 33.3 | 60 | 37.5 | 33.3 |
|  | Critical | 66.7 | 40 | 56.2 | 66.7 |
| Ability to do those things that makes one feel valuable | Not important | 0 | 0 | 6.2 | 0 |
|  | Important | 11.1 | 40 | 25 | 66.7 |
|  | Critical | 88.9 | 60 | 68.8 | 33.3 |
| Ability to experience one's life as meaningful | Not important | 0 | 0 | 6.2 | 0 |
|  | Important | 0 | 13.3 | 6.2 | 33.3 |
|  | Critical | 100 | 86.7 | 87.5 | 66.7 |
| Ability to find solutions to problems that arise | Not important | 0 | 0 | 0 | 0 |
|  | Important | 33.3 | 26.7 | 50 | 50 |
|  | Critical | 66.7 | 73.3 | 50 | 50 |
| Ability to set and reach goals | Not important | 0 | 0 | 6.2 | 16.7 |
|  | Important | 44.4 | 26.7 | 50 | 16.7 |
|  | Critical | 55.6 | 73.3 | 43.8 | 66.7 |
| Ability to achieve results and success | Not important | 0 | 0 | 26.7 | 16.7 |
|  | Important | 62.5 | 66.7 | 60 | 50 |
|  | Critical | 37.5 | 33.3 | 13.3 | 33.3 |
| Ability to be able to think about the future without concern | Not important | 0 | 20 | 12.5 | 16.7 |
|  | Important | 44.4 | 53.3 | 68.8 | 66.7 |
|  | Critical | 55.6 | 26.7 | 18.8 | 16.7 |
| Ability to effectively use coping strategies when faced with adversity | Not important | 12.5 | 0 | 0 | 16.7 |
|  | Important | 12.5 | 26.7 | 25 | 33.3 |
|  | Critical | 75 | 73.3 | 75 | 50 |
| Ability to be able to recover from adversity or trauma | Not important | 11.1 | 6.7 | 6.2 | 0 |
|  | Important | 0 | 46.7 | 43.8 | 66.7 |
|  | Critical | 88.9 | 46.7 | 50 | 33.3 |
| Ability to experiencing love, friendship, and support | Not important | 0 | 6.7 | 0 | 16.7 |
|  | Important | 11.1 | 26.7 | 31.2 | 33.3 |
|  | Critical | 88.9 | 66.7 | 68.8 | 50 |
| Ability to feel happiness and satisfaction | Not important | 0 | 0 | 0 | 16.7 |
|  | Important | 22.2 | 40 | 43.8 | 33.3 |
|  | Critical | 77.8 | 60 | 56.2 | 50 |
| *Feeling of being seen as a person who is both capable and vulnerable* | Not important | 0 | 7.1 | 0 | 16.7 |
|  | Important | 12.5 | 7.1 | 26.7 | 33.3 |
|  | Critical | 87.5 | 85.7 | 73.3 | 50 |
| Care Process Outcomes | | | | | |
| Outcomes | Scoring Category | Patient / Patient Representative (%) | Health Worker (%) | Researcher (%) | Managerial Decision-Maker (%) |
| Preconditions to plan care based on the patient's lived experience and needs | Not important | 0 | 0 | 0 | 0 |
|  | Important | 0 | 26.7 | 12.5 | 33.3 |
|  | Critical | 100 | 73.3 | 87.5 | 66.7 |
| Preconditions to adapt planning based on the patient's needs | Not important | 0 | 0 | 0 | 0 |
|  | Important | 0 | 6.7 | 6.2 | 16.7 |
|  | Critical | 100 | 93.3 | 93.8 | 83.3 |
| Ability to adapt healthcare based on goals set together with the patient | Not important | 0 | 0 | 0 | 0 |
|  | Important | 11.1 | 13.3 | 6.2 | 0 |
|  | Critical | 88.9 | 86.7 | 93.8 | 100 |
| Conditions for the patient to have their questions and concerns addressed | Not important | 0 | 0 | 0 | 0 |
|  | Important | 11.1 | 6.7 | 12.5 | 16.7 |
|  | Critical | 88.9 | 93.3 | 87.5 | 83.3 |
| Conditions for significant others to have their questions and concerns addressed | Not important | 0 | 0 | 0 | 16.7 |
|  | Important | 11.1 | 26.7 | 43.8 | 50 |
|  | Critical | 88.9 | 73.3 | 56.2 | 33.3 |
| Preconditions to be notice changes in the patient's needs | Not important | 0 | 0 | 0 | 0 |
|  | Important | 0 | 6.7 | 12.5 | 0 |
|  | Critical | 100 | 93.3 | 87.5 | 100 |
| Conditions of health care to contribute to the patient having control over the ongoing care process | Not important | 0 | 0 | 0 | 0 |
|  | Important | 22.2 | 40 | 12.5 | 16.7 |
|  | Critical | 77.8 | 60 | 87.5 | 83.3 |
| Patient experience | Not important | 11.1 | 0 | 0 | 0 |
|  | Important | 11.1 | 0 | 12.5 | 33.3 |
|  | Critical | 77.8 | 100 | 87.5 | 66.7 |
| Access to healthcare that meets everyone’s needs | Not important | 11.1 | 6.7 | 12.5 | 16.7 |
|  | Important | 0 | 40 | 43.8 | 33.3 |
|  | Critical | 88.9 | 53.3 | 43.8 | 50 |
| Accessibility to different types of healthcare | Not important | 11.1 | 20 | 6.2 | 16.7 |
|  | Important | 33.3 | 6.7 | 56.2 | 50 |
|  | Critical | 55.6 | 73.3 | 37.5 | 33.3 |
| *The patient feels they are treated with respect as an equal* | Not important | 11.1 | 6.7 | 0 | 0 |
|  | Important | 11.1 | 6.7 | 13.3 | 33.3 |
|  | Critical | 77.8 | 86.7 | 86.7 | 66.7 |
| **Possibility to choose a healthcare contact** | Not important | 11.1 | 13.3 | 12.5 | 16.7 |
|  | Important | 44.4 | 60 | 87.5 | 66.7 |
|  | Critical | 44.4 | 26.7 | 0 | 16.7 |
| *The patient's perspective and, if required significant others' perspectives, are take into account in all decisions regarding future care.* | Not important | 0 | 13.3 | 0 | 0 |
|  | Important | 11.1 | 13.3 | 31.2 | 16.7 |
|  | Critical | 88.9 | 73.3 | 68.8 | 83.3 |
| *The patient and, if required significant others', are prepared for what is to come* | Not important | 0 | 0 | 0 | 0 |
|  | Important | 11.1 | 6.7 | 12.5 | 0 |
|  | Critical | 88.9 | 93.3 | 87.5 | 100 |
| *Continuity in healthcare* | Not important | 0 | 0 | 0 | 0 |
|  | Important | 11.1 | 33.3 | 31.2 | 0 |
|  | Critical | 88.9 | 66.7 | 68.8 | 100 |
| *Ability to see the unique individual* | Not important | 0 | 6.7 | 0 | 0 |
|  | Important | 22.2 | 20 | 18.8 | 33.3 |
|  | Critical | 77.8 | 73.3 | 81.2 | 66.7 |
| Organisational Outcomes | | | | | |
| Outcomes | Scoring Category | Patient / Patient Representative (%) | Health Worker (%) | Researcher (%) | Managerial Decision-Maker (%) |
| *Health worker's ability to work independently based on their own competencies* | Not important | 11.1 | 6.7 | 0 | 0 |
|  | Important | 22.2 | 33.3 | 25 | 33.3 |
|  | Critical | 66.7 | 60 | 75 | 66.7 |
| Health worker's resource allocation | Not important | 12.5 | 0 | 0 | 0 |
|  | Important | 50 | 26.7 | 56.2 | 0 |
|  | Critical | 37.5 | 73.3 | 43.8 | 100 |
| Health worker's workload | Not important | 11.1 | 0 | 0 | 0 |
|  | Important | 33.3 | 13.3 | 37.5 | 16.7 |
|  | Critical | 55.6 | 86.7 | 62.5 | 83.3 |
| Health worker's work environment | Not important | 0 | 0 | 0 | 0 |
|  | Important | 55.6 | 13.3 | 18.8 | 0 |
|  | Critical | 44.4 | 86.7 | 81.2 | 100 |
| Health worker's ethical stress | Not important | 0 | 0 | 0 | 0 |
|  | Important | 55.6 | 13.3 | 18.8 | 16.7 |
|  | Critical | 44.4 | 86.7 | 81.2 | 83.3 |
| *Healthcare's ability to collaborate externally* | Not important | 0 | 0 | 0 | 0 |
|  | Important | 12.5 | 20 | 31.2 | 33.3 |
|  | Critical | 87.5 | 80 | 68.8 | 66.7 |
| *Healthcare's ability to collaborate internally* | Not important | 0 | 0 | 0 | 0 |
|  | Important | 0 | 6.7 | 12.5 | 16.7 |
|  | Critical | 100 | 93.3 | 87.5 | 83.3 |
| Economic Outcomes | | | | | |
| Outcomes | Scoring Category | Patient / Patient Representative (%) | Health Worker (%) | Researcher (%) | Managerial Decision-Maker (%) |
| Patient's expenses for healthcare and medication | Not important | 0 | 6.7 | 12.5 | 16.7 |
|  | Important | 44.4 | 53.3 | 68.8 | 50 |
|  | Critical | 55.6 | 40 | 18.8 | 33.3 |
| Society's costs for healthcare | Not important | 11.1 | 0 | 0 | 0 |
|  | Important | 77.8 | 20 | 43.8 | 0 |
|  | Critical | 11.1 | 80 | 56.2 | 100 |
| Society's costs for medications | Not important | 11.1 | 0 | 0 | 0 |
|  | Important | 66.7 | 46.7 | 62.5 | 16.7 |
|  | Critical | 22.2 | 53.3 | 37.5 | 83.3 |
| Patient's / service user's expenses municipal care (nursing homes and home help services) | Not important | 11.1 | 0 | 12.5 | 16.7 |
|  | Important | 33.3 | 64.3 | 50 | 33.3 |
|  | Critical | 55.6 | 35.7 | 37.5 | 50 |
| Society's costs for municipal care (nursing homes and home help services) | Not important | 11.1 | 0 | 0 | 0 |
|  | Important | 44.4 | 57.1 | 56.2 | 16.7 |
|  | Critical | 44.4 | 42.9 | 43.8 | 83.3 |
| The impact of illness on the patient's working hours or income | Not important | 0 | 6.7 | 6.2 | 16.7 |
|  | Important | 55.6 | 60 | 56.2 | 50 |
|  | Critical | 44.4 | 33.3 | 37.5 | 33.3 |
| The impact on significant others’ working hours or income due to the patient's illness | Not important | 0 | 6.7 | 12.5 | 0 |
|  | Important | 50 | 53.3 | 62.5 | 66.7 |
|  | Critical | 50 | 40 | 25 | 33.3 |
| Leisure time the patient allocates to care or self-care | Not important | 0 | 6.7 | 6.2 | 16.7 |
|  | Important | 50 | 80 | 81.2 | 50 |
|  | Critical | 50 | 13.3 | 12.5 | 33.3 |
| Leisure time the significant others allocate to patient care or self-care | Not important | 0 | 13.3 | 12.5 | 16.7 |
|  | Important | 44.4 | 66.7 | 68.8 | 66.7 |
|  | Critical | 55.6 | 20 | 18.8 | 16.7 |
| Time spent and travel costs to/from healthcare | Not important | 0 | 26.7 | 6.2 | 0 |
|  | Important | 66.7 | 46.7 | 93.8 | 66.7 |
|  | Critical | 33.3 | 26.7 | 0 | 33.3 |
| *Resource use for patient-facing work* | Not important | 0 | 0 | 7.7 | 0 |
|  | Important | 71.4 | 33.3 | 53.8 | 33.3 |
|  | Critical | 28.6 | 66.7 | 38.5 | 66.7 |
| *Resource use for administration* | Not important | 0 | 0 | 6.7 | 0 |
|  | Important | 100 | 40 | 53.3 | 33.3 |
|  | Critical | 0 | 60 | 40 | 66.7 |
| Ehealth Outcomes | | | | | |
| Outcomes | Scoring Category | Patient / Patient Representative (%) | Health Worker (%) | Researcher (%) | Managerial Decision-Maker (%) |
| Access to health information | Not important | 11.1 | 6.7 | 0 | 16.7 |
|  | Important | 22.2 | 40 | 31.2 | 16.7 |
|  | Critical | 66.7 | 53.3 | 68.8 | 66.7 |
| Accessible health information | Not important | 11.1 | 0 | 0 | 0 |
|  | Important | 33.3 | 53.3 | 31.2 | 0 |
|  | Critical | 55.6 | 46.7 | 68.8 | 100 |
| The experience of safety in the handling of personal information | Not important | 0 | 6.7 | 0 | 16.7 |
|  | Important | 22.2 | 33.3 | 31.2 | 50 |
|  | Critical | 77.8 | 60 | 68.8 | 33.3 |
| Experiencing services and functions as meaningful | Not important | 0 | 0 | 0 | 0 |
|  | Important | 11.1 | 13.3 | 31.2 | 16.7 |
|  | Critical | 88.9 | 86.7 | 68.8 | 83.3 |
| Experiencing the system as reliable | Not important | 0 | 6.7 | 0 | 0 |
|  | Important | 22.2 | 26.7 | 31.2 | 0 |
|  | Critical | 77.8 | 66.7 | 68.8 | 100 |

# **Table S4. Rating Changes Between Round One and Round Two**

| **Stakeholder Group** | **Patient / Patient Representative (n)** | **Health Worker (n)** | **Researcher (n)** | **Managerial Decision-Maker (n)** |
| --- | --- | --- | --- | --- |
| **Rating Changes** |  |  |  |  |
| 1-3 to 4-6, | 1 | 7 | 7 | 6 |
| 1-3 to 7-9 | 0 | 1 | 0 | 0 |
| 4-6 to 1-3 | 0 | 4 | 0 | 0 |
| 4-6 to 7-9 | 13 | 18 | 25 | 15 |
| 7-9 to 1-3 | 0 | 2 | 0 | 0 |
| 7-9 to 4-6 | 4 | 13 | 23 | 8 |
| Missing to 1-3 | 2 | 0 | 0 | 0 |
| Missing to 4-6 | 1 | 0 | 3 | 6 |
| Missing to 7-9 | 0 | 0 | 0 | 0 |
|  |  |  |  |  |
